# The haybiome: Characterising the viable bacterial community profile of four different hays for horses following different pre-feeding regimens

**DOI:** 10.1371/journal.pone.0242373

**Published:** 2020-11-17

**Authors:** Simon Daniels, Jacob Hepworth, Meriel Moore-Colyer

**Affiliations:** School of Equine Management and Science, Royal Agricultural University, Cirencester, Gloucestershire, United Kingdom; Osmania University, INDIA

## Abstract

Respirable dust in conserved forages can pose problems with equid respiratory health, thus soaking (W) and high temperature steaming (HTS) are employed to reduce the levels in hay. The aim of this study was to characterize the viable bacterial community profile of four hays from two different locations in UK following pre-feeding wetting regimens. Hypothesis: (1) Viable microbial community profile of hays will not differ between different pre-feeding regimens. (2) Hay type and location will not influence microbial community profile. Replicates of each of the four hays were subjected to dry (D), HTS conducted in a HG600, W by submergence in 45 L tap water, 16°C for 12 hours. From each post-treated hay, 100 g samples were chopped and half (n = 36) treated with Propidium monoazide dye, the remaining half untreated. Bacterial DNA were extracted for profiling the V4-V5 region of 16S rRNA gene from all 72 samples, then sequenced on the Illumina MiSeq platform. Bioinformatics were conducted using QIIME pipeline (v1.9.1). Linear discriminate analysis effect size (LEfSe) was used to identify differences in operational taxonomic units and predicted metabolic pathways between hays and regimens. HTS reduced proportions of microbiota compared to W and D hay (P < 0.001, df 3, F 13.91), viability was reduced within regimens (P = 0.017, df 1, F 5.73). Soaking reduced diversity within and between regimens. Core bacterial communities differed between hays and regimens, however pre-feeding regimen had the greatest effect on the bacterial community profile. W and HTS reduced viable bacteria (P< 0.05) known to cause respiratory disease, for HTS both respiratory and dental disease, with the greatest reductions overall from HTS without reducing bacterial diversity. Soaking increased Gram-negative bacteria and reduced bacterial diversity. Collectively these findings add to a body of evidence that suggest HTS is the most suitable pre-feeding regimen of hay for equid health.

## Introduction

Conserved forages are commonly fed to horses’ at times where fresh forage is unavailable [1]. Hay is the predominant forage source fed to horses’ in the UK [2], however the hygienic quality of hay is inherently variable and has been linked to sward composition grassland management, conservation techniques and environmental conditions, all of which can play a role in the quality of the forage [3, 4]. When feeding, horses’ inhale aeroallergens from forage which can exacerbate respiratory diseases. To reduce aeroallergens different wetting treatments have been used and proven to be highly effective [5–7]. However soaking in water for 30 minutes or more has some well documented disadvantages such as losses of P, K, Mg, Na and Cu. Additional nutrient losses from soaking include water soluble carbohydrates (WSC) and a reduction in overall dry matter [8, 9]. Following soaking the water left behind has been reported to have a biological oxygen demand, a common marker of water pollution, 10 times greater than raw sewage [10].

Culturing has shown that soaking hay increases bacterial concentrations [7], but the bacterial profile and the effect these bacteria might have on the horses’ gastrointestinal tract are currently unknown. More recently high temperature steaming (HTS) has been used to reduce aeroallergens in hay, Moore-Colyer *et al*. [7] showed that steaming reduced the bacterial, yeast and fungal concentrations of hays when compared with soaking. These authors also found that steaming did not alter the mineral or protein profile of the hays and losses of WSC were highly variable ranging from 2–50%. In the same study Moore-Colyer *et al*. [7] showed a 100% reduction in fungal growth following HTS which is highly beneficial in preventing respiratory infection. While HTS has been shown to reduce bacteria, some bacteria still survive the process but these have not all been successfully cultured and identified [7, 11].

A recent study in humans identified that microbiota found in food were also identified in faeces [12]. Salem *et al*. [13] postulate that if this occurs in humans it is plausible that the bacteria on forage may influence the equid gastrointestinal microbiome. Thus it is important to understand the effects any pre-feeding regimens might have on the microbiome of the forage itself. More recently Moore-Colyer *et al*. [14] characterized the composition of microbiota present on three hays when dry and following differing soaking regimens. These authors reported that soaking for up to 16 hours altered bacterial phyla including Proteobacteria, Cyanobacteria and Deferribacteres. Of these phyla, none play key roles in fibre hydrolysis within the gut, however each phyla also contain pathogenic bcteria associated with intestinal disease alongside non-pathogenic species [15, 16]. The findings of Moore-Colyer *et al*. [14] provide a valuable insight into the microbiota composition of hays following soaking, however this study does not consider HTS nor does it consider the viability of microbiota found on the forage. To the authors’ knowledge, this is the first study of its kind aiming to characterize the viable bacterial community profile of dry, soaked and HTS hay that we refer to as the haybiome.

The objective of this study was to characterize the bacterial community profile of four different hays from two different locations in UK and to identify viable bacteria when dry, following high temperature steaming or soaking.

Hypothesis: (1) The viable microbial community profile of hays will not differ between different pre-feeding regimens. (2) Hay type and location will not influence microbial community profile.

## Materials and methods

This study was approved by the RAU research ethics committee.

### Hays

Four different hays, two Meadow hays (M) and two Italian ryegrass (IRG) hays, were sourced from two different locations, (1) Banbury, Oxfordshire and (2) Slimbridge, Gloucestershire in the South West of England. Both farms were commercial forage enterprises selling hay, replicate bales of M and IRG, three small bales (approx. 22 kg) were collected for each hay, from each supplier, thus 3xM and 3xIRG from location one and 3xM and 3xIRG from location two. Hays were harvested during the summer of 2017, data on the agricultural practices employed were collected from each supplier. IRG hays were a mono culture and produced from short term leys with an average of four days between cutting and baling. Meadow hays were from permanent pastures, containing a mixture of species including; meadow grasses, perennial ryegrass, crested dogs tail and Yorkshire fog. Meadow hays were cut and baled within five days. Location two reported some rainfall during the hay making of both the IRG and M hays. Both locations barn stored their hays for six months prior to selling and made use of drying floors, whereby the hay was subject to further drying in the barn following harvest.

### Sampling

Each of the four hays were processed individually as follows: The three replicate bales were opened on a new clean plastic sheet and thoroughly mixed using gloved hands. Nine 2.5 kg hay nets (3 replicates per regimen) were compiled from the approx. 66 kg of each hay, thus the hay nets comprised 34% of the total of each hay that was sourced.

### Pre-feeding regimens

Each of the 36 replicate hay nets (4 hays, 3 reps per hay, 3 regimens) were subjected to one of the following regimens: (1) Dry (D) remained untreated (control). (2) Soaking (W): each net was fully immersed and weighed down, in 45 L of tap water at 16°C for 12 hours. Soaking time was based upon practical application and common soak lengths employed by horse managers [11] to reduce WSC, and the findings of altered microbiota abundance on hays post soaking from Moore-Colyer *et al*. [14]. Post treatment hay nets were hung up to drain for 10 minutes as per the procedure of Moore-Colyer *et al*. [11] before being sub-sampled. (3) Steamed (HTS) hay nets were placed into a HG 600 (Haygain Ltd) and fully pressed down onto the spiked manifold. Wooden rulers with non-reversible temperature strips (555–409, RoHS Scale B Self-adhesive, testo, www.testo.com) were pushed into the hay net and the lid closed so the hay was sealed inside the insulated chest. The boiler was filled with 7 liters of clean tap water and turned on. Steaming took approx. 60 minutes and following the manufacturer’s instructions, which detailed that the steamer temperature gauge on the lid should read 80°C for a minimum of 10 minutes. This ensured that all the hay was thoroughly steamed and that the temperature inside the hay was in excess of 95°C for a minimum of 10 minutes, which was verified by the temperature strips inside the hay.

Following each of the regimens the contents of the hay net was emptied onto a clean plastic sheet and grab samples, using gloved hands, taken in a W formation making up a 100 g sub sample which was used for DNA extraction from each hay type.

### PMA treatment

Each of the 100 g hay sub-samples were split into two (50 g), half of the samples (n = 36) were further sub-sampled for 1 g to be treated with Propidium monoazide (PMA) dye (Biotium inc). PMA dye exposure to dead and inviable bacteria binds to DNA and when fluoresced photo activation alters the DNA structure preventing replication during polymerase chain reaction, thus leaving only live viable bacteria in the sample [17]. The remaining (50 g) (n = 36) were left untreated so the total combination of dead and viable bacterial DNA could be amplified by viable polymerase chain reaction (vPCR) for sequencing, thus in total 72 samples were prepared for 16S rRNA sequencing. Samples were chopped using a sterile scalpel. For PMA treatment, 1 g of sample was subjected to 5 ml of a 50 μmol solution of PMA dissolved in dimethyl sulfoxide. Samples were briefly vortexed and incubated in the dark for 5 minutes. Following incubation, samples were placed on ice in a tray and fluoresced under a 650w halogen lamp for 10 minutes as described by Tian *et al*. [18]. Following this samples were filtered using Whatman 541 filter paper and hay collected for bacterial DNA extraction.

### DNA extraction

Bacterial DNA were extracted using the DNeasy PowerMax® soil kit, following the manufacturer’s instructions. DNA concentration was confirmed in all samples by Nano drop ND-1000 spectrometer prior to PCR. A sub sample (n = 18) were also analysed for DNA content by gel electrophoresis.

### Bacterial community profiling

Pre-sequencing, aliquots of extracted DNA were amplified with universal primers for the V4 and V5 regions of the 16S rRNA gene using primers U515F (5’-GTGYCAGCMGCCGCGGTA) and U927R (5’-CCCGYCAATTCMTTTRAGT) [19]. Amplicons were purified using 0.8 volumes of Ampure XP magnetic beads (Beckman Coulter). Each sample was then tagged with a unique pair of indices and the sequencing primer, using Nextera XT v2 Index kits, and 2x KAPA HiFi HotStart ReadyMix using the following cycling conditions: 95°C for 3 min; 10 cycles of 95°C for 30 s, 55°C for 30 s, 72°C for 30 s; followed by 72°C for 5 min. Index-tagged amplicons were purified using 0.8 volumes of Ampure XP magnetic beads (Beckman Coulter). The concentration of each sample was measured using the fluorescence-based Quantifluor assay (Promega). Concentrations were normalized before pooling all samples, each of which would be subsequently identified by its unique index combination. Sequencing was performed on an Illumina MiSeq with 2 × 300 base reads according to the manufacturer’s instructions (Illumina Cambridge UK) by the Animal and Plant Health Agency. Bioinformatics were conducted using the QIIME pipeline (Version 1.9.1) [20], in the Microbiome Helper package [21]. Sequences were demultiplexed and then quality checked using FastQC [22]. Chimeric reads were then filtered out using VSEARCH [23]. Sequences clustered and assigned to operational taxonomic units (OTUs) of 97% similarity using open reference OTU picking combining SortMeRNA [24] and SUMACLUST [25]. The OTU table was not rarefied to prevent loss of valuable data [26]. The OTU table was then used for further downstream analysis, rarefaction analysis and calculation of diversity indices were conducted using QIIME, alpha diversity was calculated using Chao 1 index and Observed species index. Beta diversity was calculated using the unweighted UniFrac method [27]. The “phylogenetic investigation of communities by reconstruction of unobserved states” (PICRUSt) platform was used to predict the metabolic function from the OTU table [28]. For this the OTU table was filtered to become closed reference using the Greengenes database (Version 13.8) [29].

### Bacterial viability

Hay samples from each pre-feeding regimen that were not treated with PMA represented both viable and non-viable bacteria, from here we classify these samples as total bacteria. Whereas those that were PMA treated are classified as containing only viable bacteria. By comparing the same hay sample following the same pre-feeding regimen with or without PMA, differences detected between these samples would be due to bacterial viability. Initially the effect of pre-feeding regimen, irrespective of hay type, was investigated on bacterial viability.

**Fig 1:**
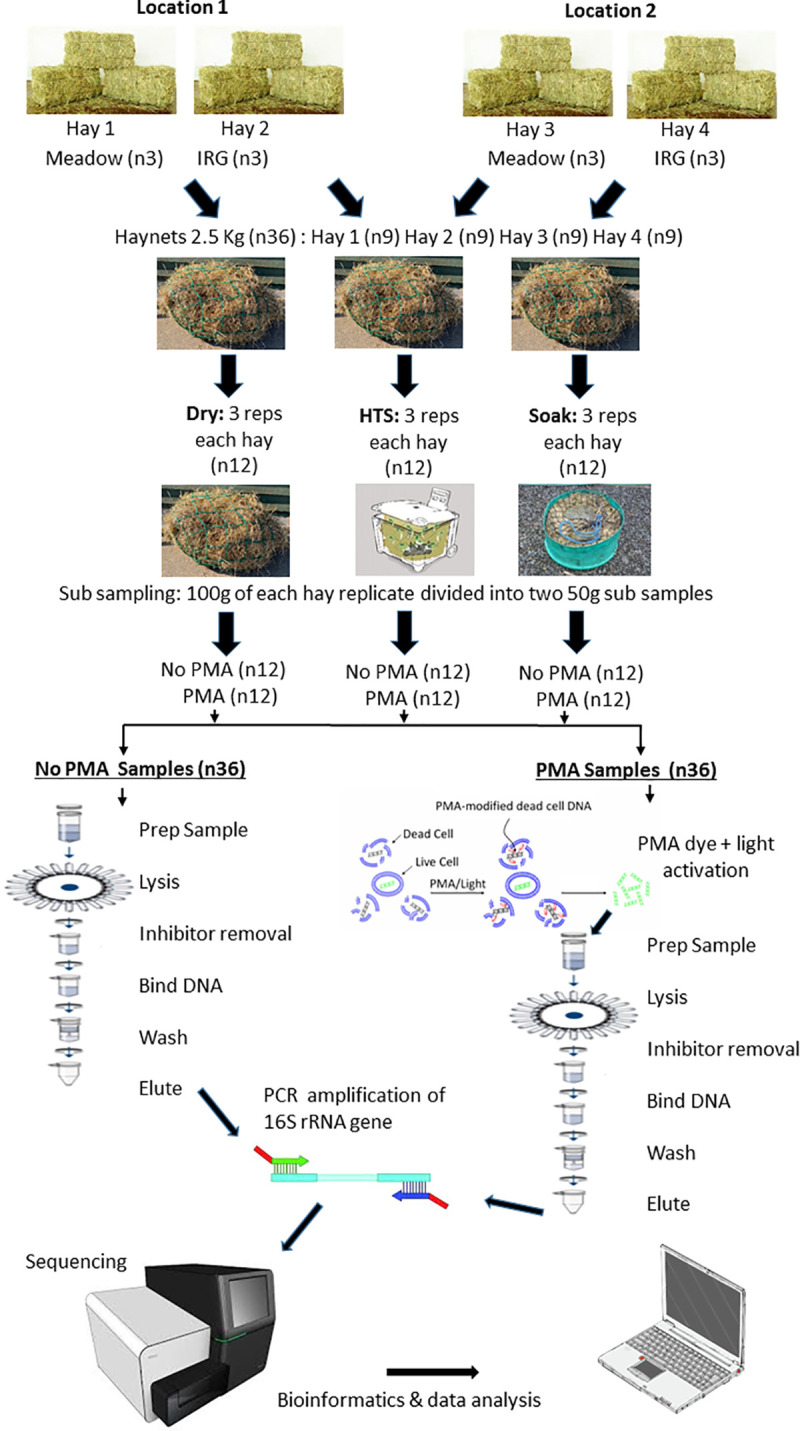
Flow diagram of the sampling method. Flow diagram of the experimental method, PMA adapted from PromoKine, DNA extraction adapted from Qiagen.

A flow diagram of the method is depicted in Fig 1.

### Data analysis

Linear discriminant analysis effect size (LEfSe) is a specific model for metagenomics data, it uses a Kruskall Wallis then Wilcoxon approach followed by effect size to give an LDA score, LDA scores can be interpreted as the degree of consistent difference in relative abundance between groups [30]. LEfSe analysis was used to identify differentially abundant OTUs from the OTU table at phyla and genera taxonomic levels between the different pre-feeding regimens. Kyoto encyclopedia of genes and genome (KEGG) orthologs, database of molecular functions and pathways, from the predicted metabolic pathways between hays and regimens predicted by PICRUSt were also analysed using LEfSe. Differences between total bacteria and viable bacteria for each regimen were analysed using the Statistical Analysis of Metagenomic Profiles package (STAMP) [31], using principal component analysis for multiple groups and using White *et al*. [32] non-parametric T-Test for two group comparisons. To identify the effect of bacterial viability on sequence read counts a generalized linear mixed effects model was used, whereby viability was nested within treatment. Post hoc 95% confidence intervals were used to detect differences between groups, Genstat 18^th^ Ed. For diversity indices alpha diversity was analysed by Kruskall-Wallis non-parametric ANOVA, Mann-Whitney *post hoc* and Bonferroni correction, conducted in Genstat 18^th^ Ed. Beta diversity was analysed using the ADONIS non-parametric ANOVA with 1000 permutations using the QIIME pipeline. Core biome community analysis, using the membership function as defined by Wang *et al*. [33], considering OTUs represented in hays and treatments, was conducted using the metagenomics core microbiome exploration tool (MetaCoMET) [33].

## Results

From the 72 samples sent for sequencing, 16 samples were rejected during data analysis due to very low reads of bacterial DNA. Of the samples 92% of the dry hay samples were retained, 75% of the soaked samples were retained and 66% of the HTS samples retained. The OTU table contained 1022 OTUs, total number of reads in the table were 1,893,567, with a mean of 33,814 reads per sample.

### Pre-feeding regimen read counts

**Fig 2:**
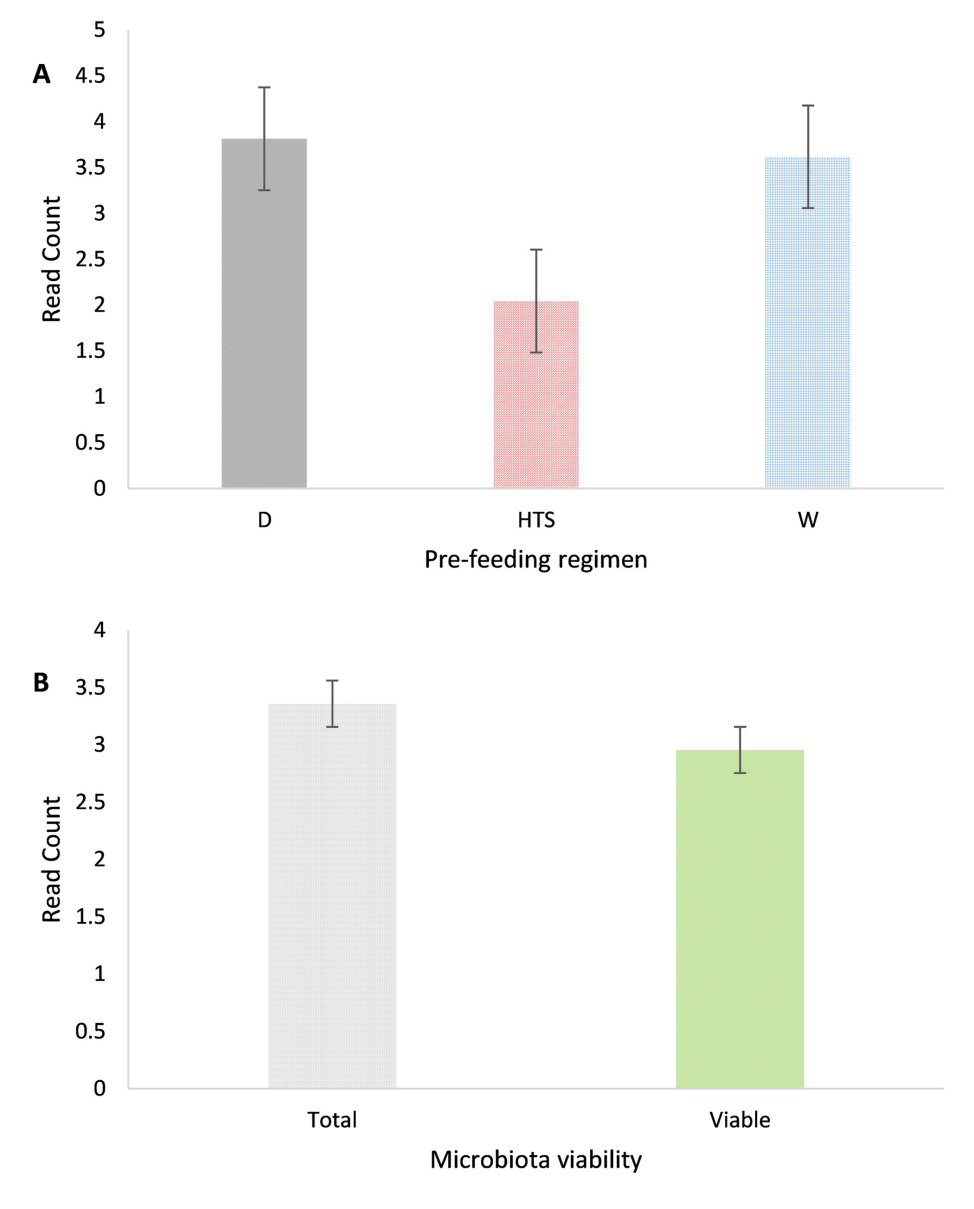
(A) Differences in sequence read counts between pre-feeding regimens (D n = 22, HTS n = 16, W n = 18) (B) total (n = 26) and viable (n = 30) counts of microbiota nested within pre-feeding regimens.

Differences in read counts were detected between the differing pre-feeding regimens (P<0.001, df. 2, F 13.91), whereby HTS had lower read counts than dry and soaked samples which were not different from each other, Fig 2A. The sub-group of viability also differed (*P =* 0.017, d.f. 1, F 5.73) with greater read counts in the total compared the viable samples, Fig 2B.

### Bacterial viability

**Fig 3:**
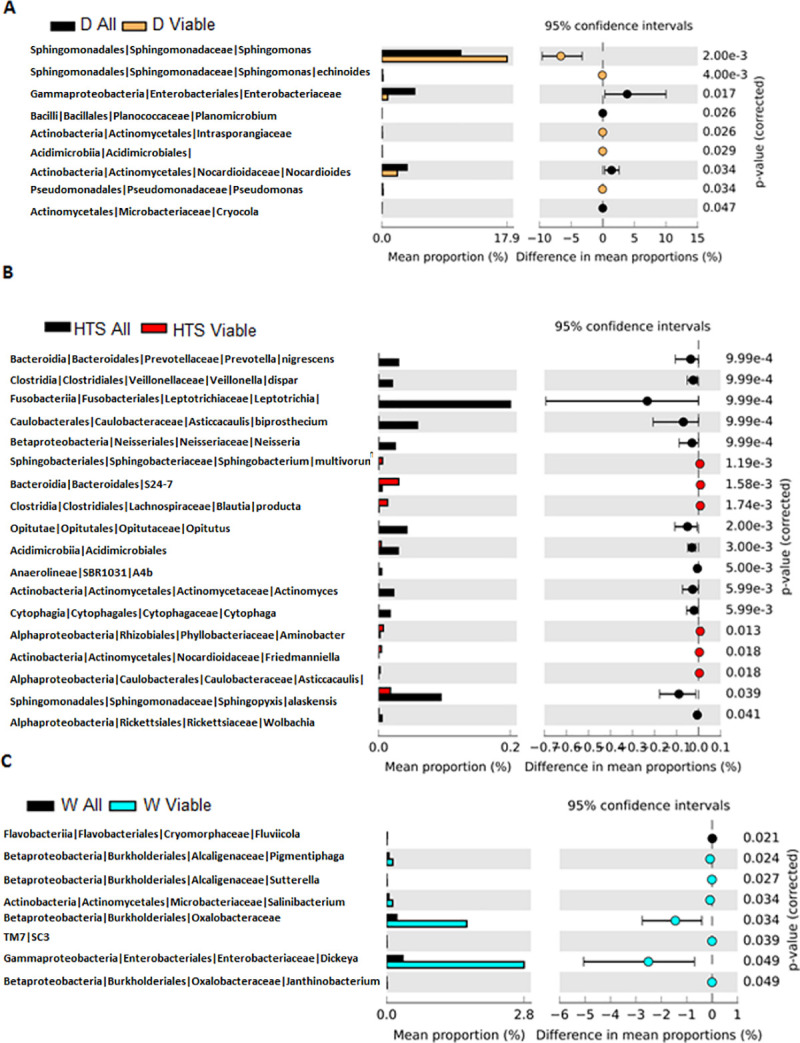
OTU viability following regimens OTUs from dry (total n = 13, viable n = 11) (A), HTS (total n = 7, viable n = 6) (B) and soaked (total n = 7, viable n = 11) (C) hay samples, all represents total bacteria and viable represents viable bacteria after PMA treatment.

Viability of bacteria did not appear to effect the alpha nor beta diversity of bacteria within or between the hay samples for each of the pre-feeding regimens, S1 Fig. This suggests that the richness within populations and the difference between microbiota populations were not altered by bacterial viability. The effect of the three pre-feeding regimens on the viability of OTUs are shown in Fig 3 and show clear treatment effects. When looking at the differences in bacterial profile between viable bacteria and total bacteria, the dry hay had nine significantly differing OTUs, four were only in the total bacteria samples and five were only in the viable samples. The proportion of these viable bacteria increased following PMA treatment which is an effect of removing the non-viable bacteria from the sample rather than an increase per *se*.

After HTS there were differences in 18 different OTUs between total and viable bacteria, there was a proportional increase in four viable OTUs and 14 total bacterial OTUs which were removed by HTS. In the soaked hay there was a reduction in only one OTU from the total bacteria samples and a proportional increase in seven OTUs in the viable samples. Comparing viable and non-viable bacteria, HTS was the most effective regimen in reducing bacterial viability as seen in Fig 3.

From the OTU table a percentage reduction in non-viable OTUs when compared to viable OTUs was calculated, S1 Table. Post treatment the HTS hay had the most OTUs that were non-viable. Of those non-viable OTUs two three are known to be linked to respiratory infections (two *Pseudomonas spp*. and *Stenotrophomonas spp)* and six are linked to oral infections and dental disease (three *Prevotella spp*., *Phorphyromonas spp*., *Fusobacterium spp*. and Actinomyes spp.). Following this stage, unless stated, all data presented are referring to viable bacteria.

### Pre-feeding regimens

**Fig 4:**
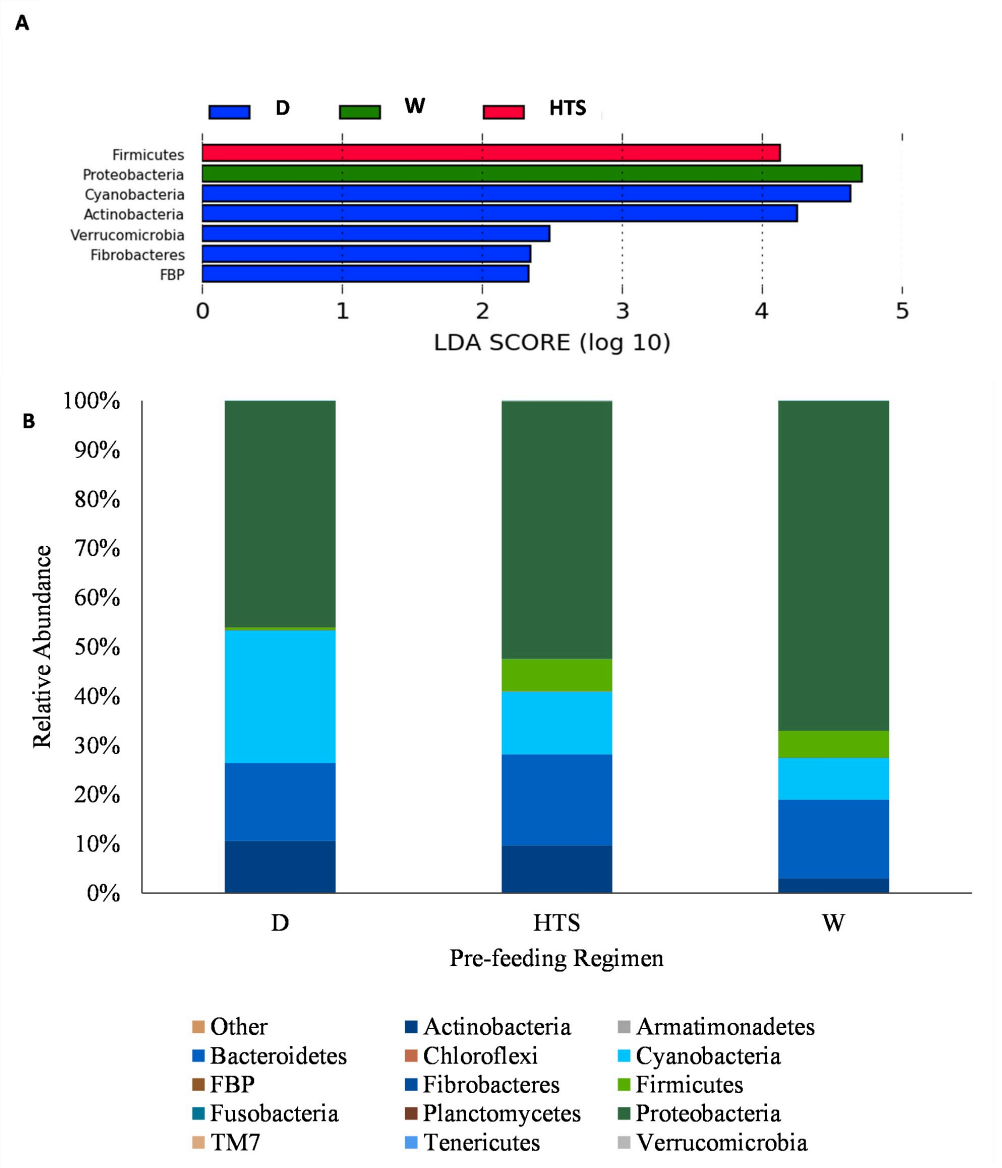
Effect of pre-feeding treatment regimens on relative abundance of bacterial phyla (A) between wetting regimens B) profile of bacterial abundance for the three different regimens, dry (D n = 10), HTS (n = 9) and soaked (W n = 11).

**Fig 5:**
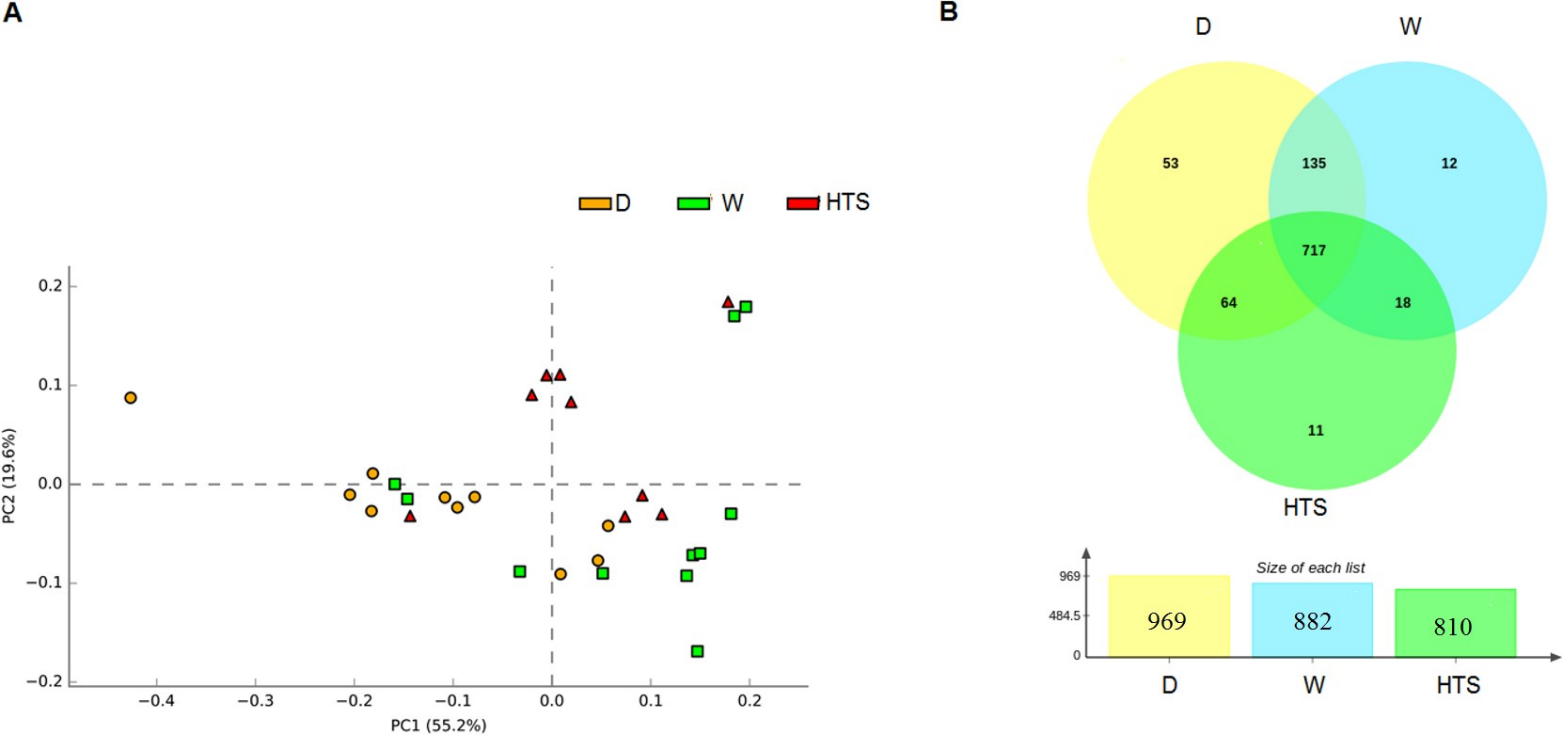
Effect of regimen on community profile of hays (A) PCA loadings plot of viable profile between regimens, notably HTS samples and (B) Venn diagram of the core community, shared and unique OTUs specific to D (n = 10), W (n = 11) and HTS (n = 9) treatments.

**Fig 6:**
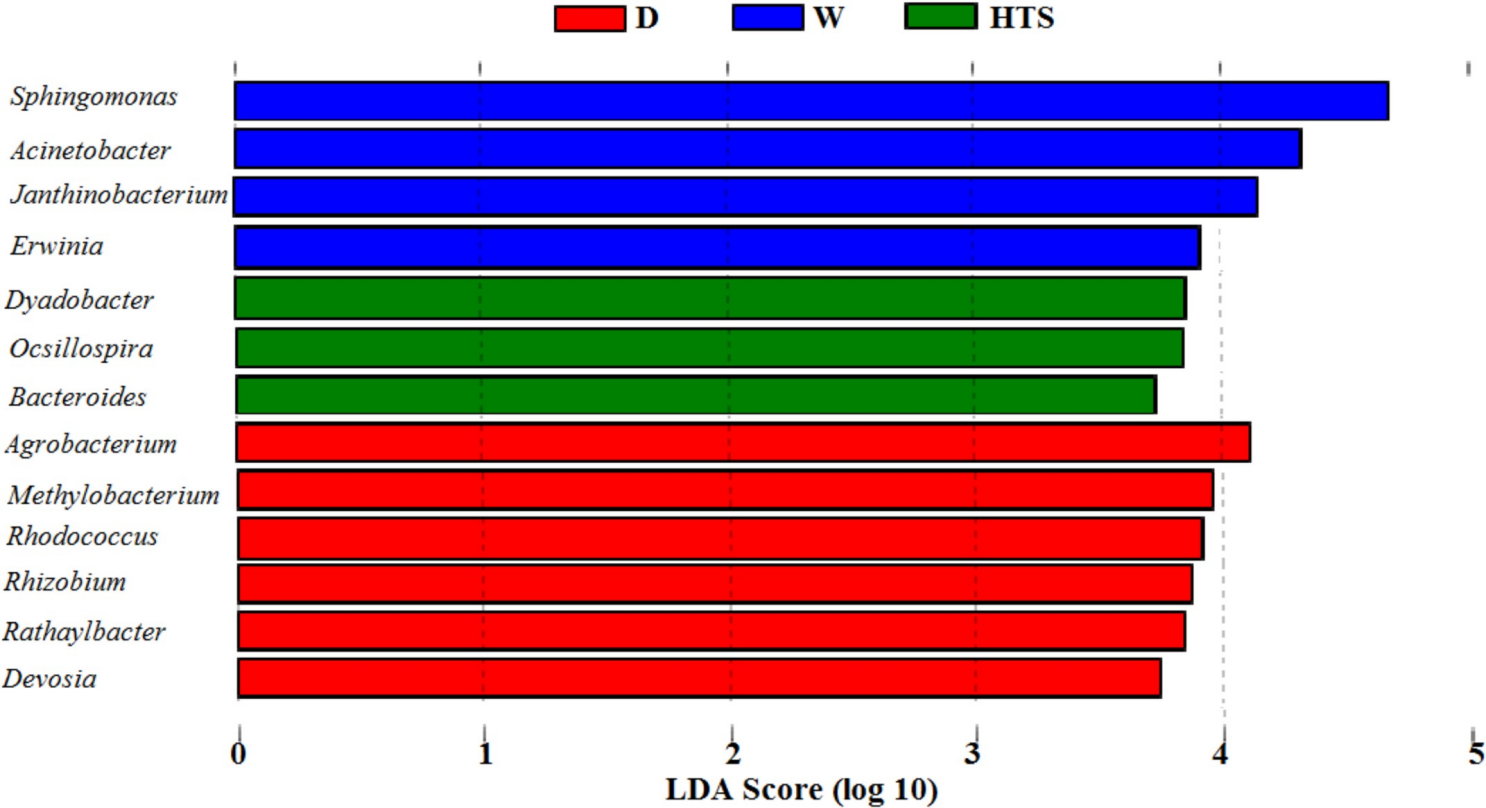
OTUs of viable bacteria differing between regimens at genera dry (D n = 10), HTS (n = 9) and soaked (W n = 11).

When considering the effect the differing regimens had on bacterial diversity and relative abundance hays were grouped by regimen irrespective of hay type and location. At Phyla dry hay showed greater abundance of Cyanobacteria (*P* = 0.01, LDA >2.0), Actinobacteria (*P* = 0.0002, LDA >2.0), Verrucmicrobia (*P* = 0.006, LDA >2.0), Fibrobacteres (*P* = 0.04, LDA >2.0) and FBP (*P* = 0.01, LDA >2.0) compared to HTS and W hays. The HTS samples showed an increased abundance of Firmicutes (*P* = 0.0003, LDA>2.0) and the soaked samples also showed an increased abundance of Proteobacteria (*P* = 0.001, LDA>2.0). These differences in relative abundance can be seen in Fig 4. Within the OTU table the effect of pre-feeding regimen can be seen in the PCA loadings plot and core bacterial community Venn diagram, Fig 5. Moving down to *genera* dry hay had 6 OTUs in greater abundance (LDA >2.0) than the HTS and soaked hays. These OTUs represented *genera* containing predominantly plant pathogens or non-pathogenic species (*Agrobacterium P* = 0.0007; *Devosia P* = 0.0007; *Methylobacterium P* = 0.004; *Rathayibacter P* = 0.001; *Rhizobium P* = 0.02), however *Rhodococcus (P* = 0.0009) contain species known to be respiratory pathogens. HTS hay had 3 OTUs in greater abundance compared to the other regimens, these *genera* included species that are non-pathogenic (*Dyadobacter P* = 0.007; *Oscillopsia P* = 0.0003). *Bacteroides* (*P* = 0.001) were also detected and this *genera* do contain some species known to cause diarrhea and lower respiratory tract infections, this *genera* also contains non-pathogenic species. Soaked hay also had 4 OTUs present in greater abundance than the other regimens, with *genera* containing known respiratory (*Acinetobacter P* = 0.03) plant (*Erwinia P* = 0.02) and fish pathogens (*Acinetobacter P* = 0.03; *Janthinobacterium P* = 0.006), Fig 6.

**Fig 7:**
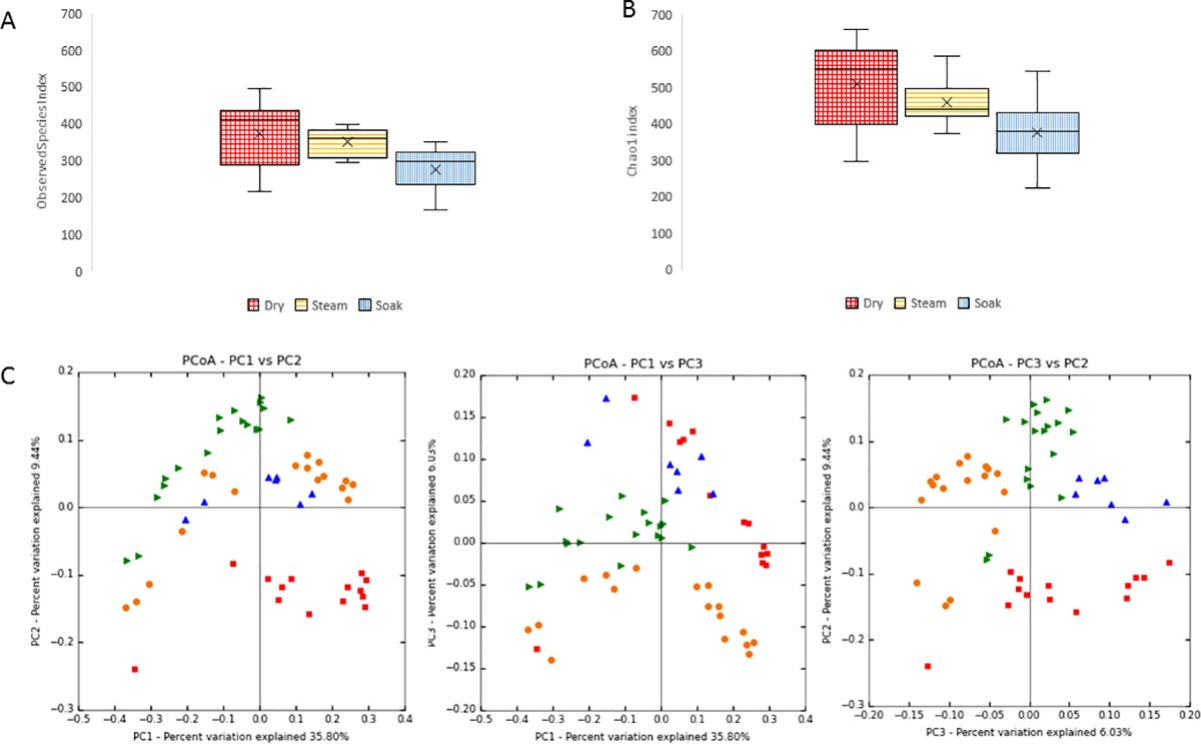
Effect of regimens on diversity, (A) Observed species (B) Chao1, 5000 seqs per sample, W (n = 11) hay was less diverse than D (n = 10) & HTS (n = 9). (C) Beta diversity between regimens PCoA loadings plots of unweighted unifrac D (n = 10), W (n = 11) and HTS (n = 9).

When considering diversity indices the soaked hay samples showed reduced alpha diversity (Observed species *P <* 0.001, df 2, H 52.07 and Chao1 *P* < 0.001, df 2, H 65.03) compared to the HTS and D samples which were not different from each other on the Chao1 or Observed species indices following post *hoc* and Bonferroni correction. Beta diversity using unweighted unifrac index also showed differences between pre-feeding regimens (*P* = 0.001, df 5, F 2.60) where D and HTS samples differed to W, Fig 7.

### Hay type & location

**Fig 8:**
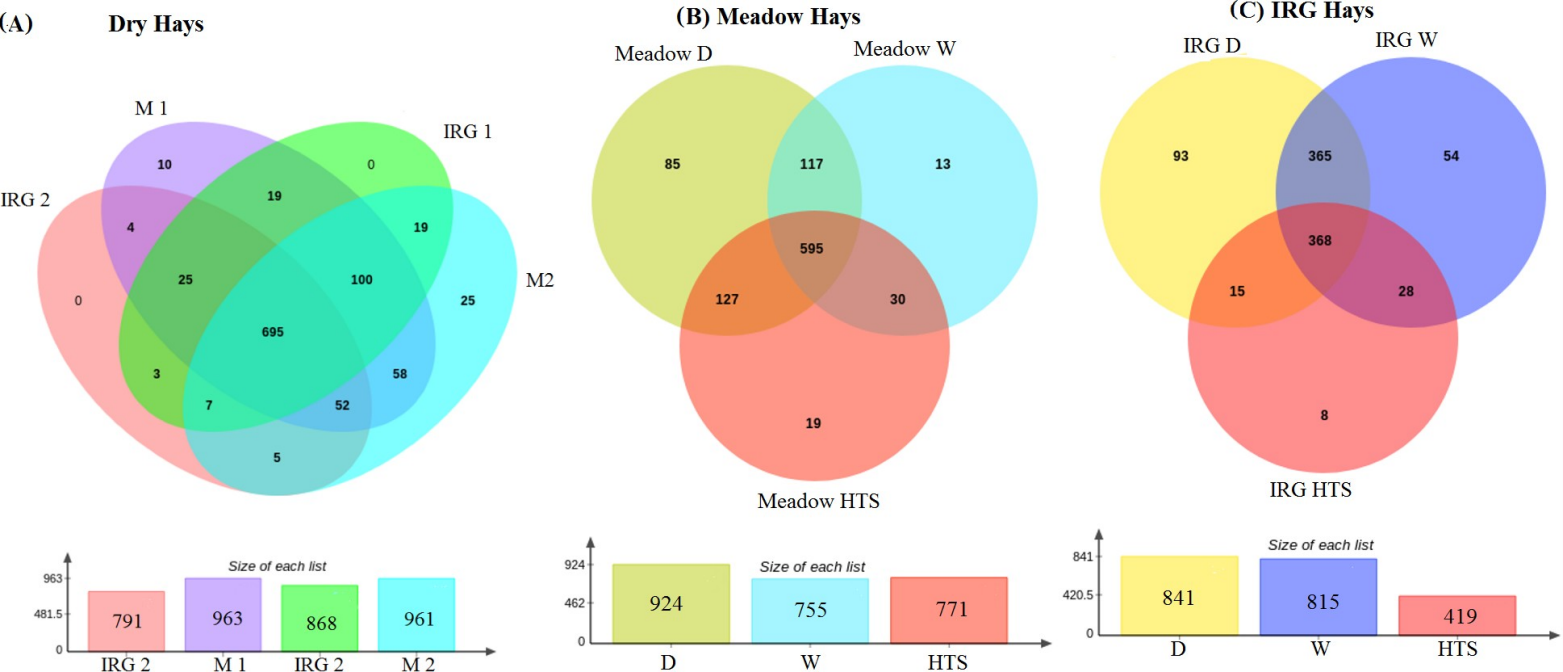
Core community for hay type, location and regimens. (A) Individual dry hays (M1 n = 3, M2 n = 3, IRG 1 n = 1, IRG2 n = 3), (B) Meadow hays following the regimens (D n = 6, HTS n = 6, W n = 6) and (C) IRG hays following regimens (D n = 4, HTS n = 3, W n = 5).

**Fig 9:**
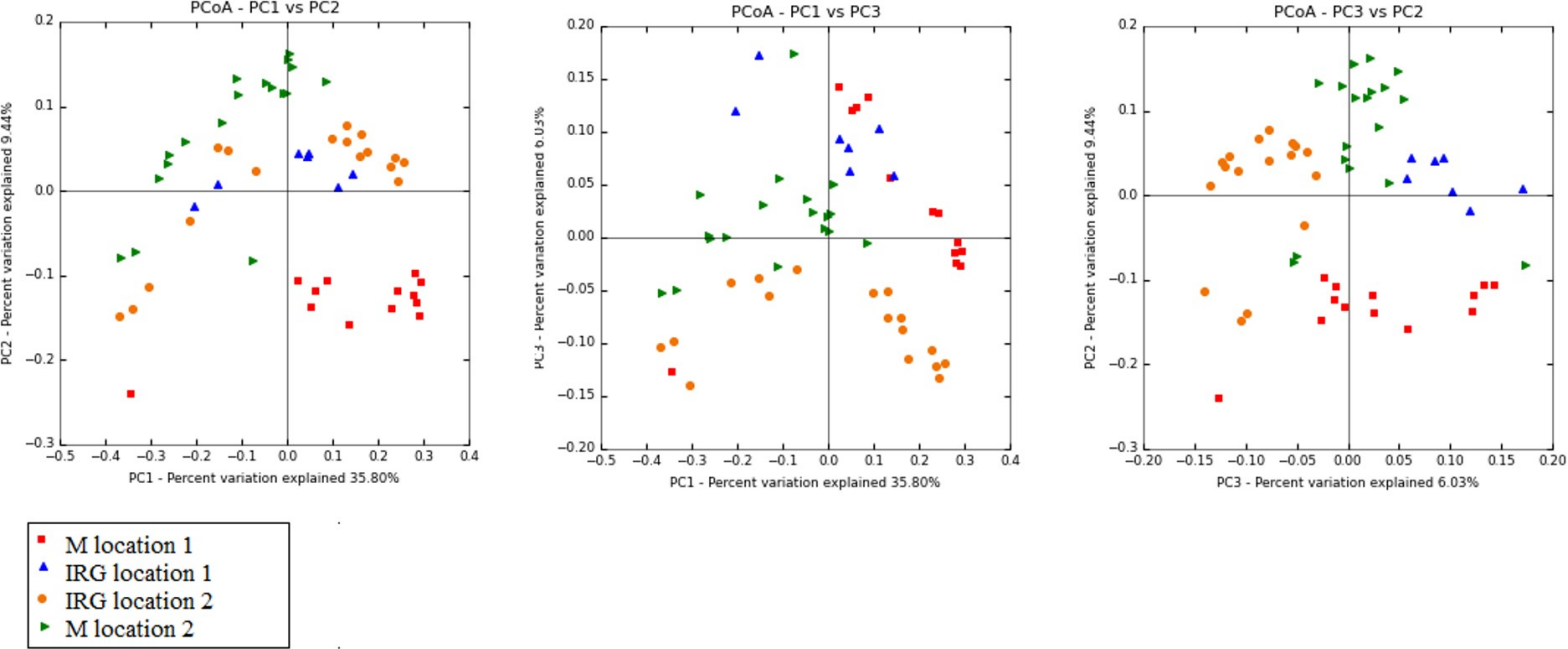
Beta diversity of the four different hays PCoA loadings plots of unweighted unifrac (M1 n = 3, M2 n = 3, IRG 1 n = 1, IRG2 n = 3). It is clear that meadow 1 is different to the other hays.

Comparing the effect of hay type and location on each dry hay, M and IRG, identified a core community and unique OTUs for each hay alongside shared OTUs between hay type and location, Fig 8. All hays shared a core community of 695 common OTUs, both of the meadow hays had 10 and 25 unique OTUs respectively, the IRG hays had no unique OTUs. The effect of location and all the other management factors may be explained by the 19 shared OTUs between M and IRG hays from location 1 and five OTUs shared between the M and IRG hays from location 2. However the core community and diversity indices showed that the effect of pre-feeding regimen was much greater than the effect of hay type or location on the bacterial community profile. The core community of the meadow hays from both locations collectively was 595 OTUs with unique OTUs for each of the pre-feeding regimens, 85 unique to D meadow hay, 19 unique to HTS meadow hay and 13 unique to soaked meadow hay. Steamed and dry meadow hay shared 127 OTUs. In the IRG hays the core community was smaller, 368 OTUs, with 93 unique to dry IRG, 54 unique to soaked IRG and eight unique to HTS IRG hay. For IRG the shared OTUs between dry and soaked hay were a similar size to the overall core community with 365 OTUs. Dry and HTS IRG hay shared 15 OTUs and HTS and soaked IRG 28 OTUs. The four individual dry hays for the two different location differed in beta diversity, Fig 9, whereby meadow hay from location1 differed in diversity to meadow hay 2 and IRG from locations 1 and 2 (*P =* 0.001, df 3, F 2.2).

### Predicted bacterial function

**Fig 10:**
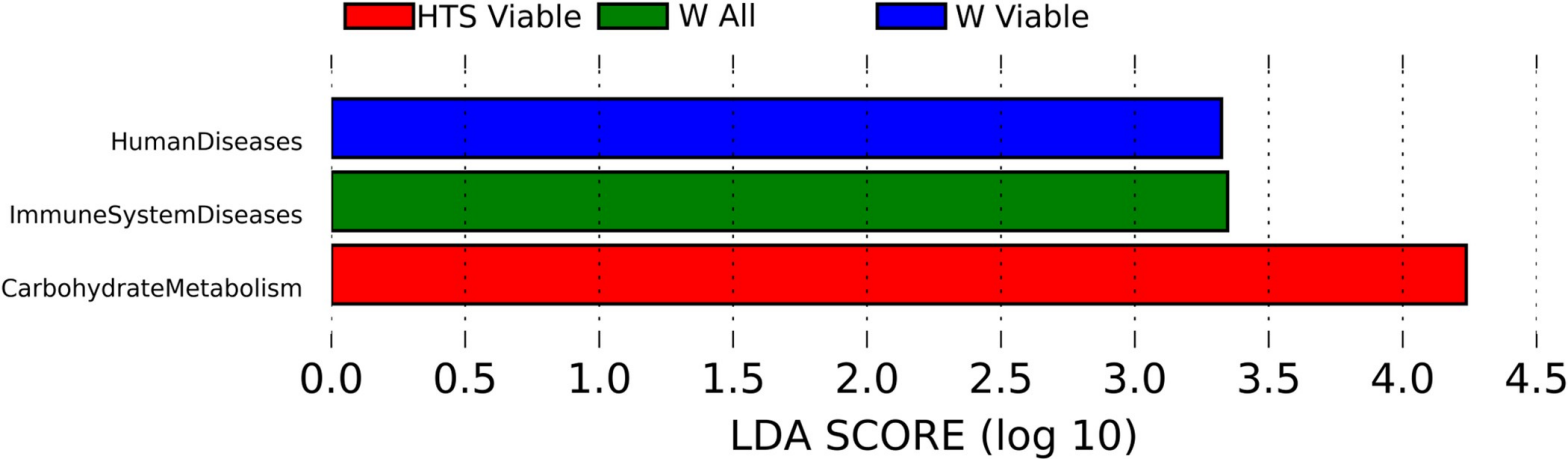
Differing KEGG orthologs between regimens level 2 KEGG othologs of predicted metabolic pathways following the different pre-feeding regimens. HTS; carbohydrate metabolism (n = 7), W; human diseases (n = 11) and immune system disease (n = 7).

From the microbial community profile, OTUs present were assigned to KEGG orthologs using the PICRUSt package. At the higher level the bacterial community in HTS hay were associated with carbohydrate metabolism (*P* = 0.01, LDA >2.0). Whereas in soaked hay communities associated with human diseases (*P* = 0.02, LDA > 2.0) and immune system disease (*P* = 0.03, LDA >2.0) were detected. However immune system diseases were detected from samples of total rather than viable bacteria, Fig 10.

## Discussion

Here we report the findings of the first study of its kind to characterise the viable bacterial community profile of hay for horses following different pre-feeding regimens’. Previous work from our group have identified that steaming hay reduces, bacterial, fungal and yeast colony forming units whereas these increase with soaking [7]. However the previous findings did not discriminate between non-viable and viable bacteria nor detailed the function of surviving bacteria. The use of the PMA dye for vPCR (which removed the non-viable bacteria) provides a novel insight into the proportion of viable to non-viable bacteria on hay following different pre-feeding regimens. The results reported here support the previous culture studies of Moore-Colyer *et al*., [7] who reported a significant reduction in total bacteria post HTS and a significant increase in total bacteria post soaking, this present study also showed significantly greater read counts in post soaked hay compared with the HTS samples.

Most of the bacteria detected from dry hay were non-pathogenic soil and plant dwelling bacteria, or those found normally within the horse’s gut microbiome. The *genera* identified following the three regimens in this study have previously been found in dry and soaked hay profiles [14]. At Phyla level dry hay showed a greater abundance of cyanobacteria (blue-green algae) which were reduced through the process of soaking and steaming. While cyanobacteria in water are a disease risk to livestock, recent work from McGorum *et al*. [16] identified that terrestrial cyanobacteria are common in low numbers on livestock fields. It is currently unknown if the toxins that cyanobacteria produce are a disease risk on forage, although the reduction seen with these wetting regimens is probably favorable for animal health. Some of the cyanobacteria detection may also represent chloroplasts from the plant cells, although these should not be present in 16S rRNA read assignments [34]. Verrucomicrobia were also present on the dry hay, these soil dwelling bacteria, of which some species commonly inhabit the horse’s large intestine and make up a part of the normal gut microbiome Fibrobacters are also an important phyla within the horse’s digestive system playing a large role in the digestion of cellulose [13, 35] were in greatest abundance in the dry hay samples. Actinobacteria, also found in greatest abundance on the dry hay, are an important group of soil dwelling bacteria also found in the gut. They possess many important properties including acting as probiotics and the production of antibiotics [36]. FBP is a division of Armatimonadetes phyla found in soil [37].

In the HTS hays there was an increase in abundance of the Firmicutes phyla. Firmicutes are the major phyla of the equid gut microbiome and play an important role in carbohydrate fermentation [13, 35]. This also corresponds to the predicted improved function of carbohydrate metabolism as identified in the predicted metabolic function, Fig 10. Dry hay had a greater abundance of Fibrobacteres, which also play a role in structural carbohydrate degradation, compared to HTS and soaked hays. However the predominant phyla for structural carbohydrate degradation in the horse are the Firmicutes [13, 35]. It is likely that the predicted differences in metabolic function were associated with a greater difference in Firmicutes (*P =* 0.0003) than Fibrobacteres (*P =* 0.04) as the assumptions of PICRUSt are based upon 16S reads. In the soaked hay there was an increase in abundance of proteobacteria, this phyla of Gram negative bacteria that include many pathogens, also play a role in nitrogen fixation. Proteobacteria are ubiquitous in drinking water [38] and commonly found in water tanks and pools thus not surprising they were found on the soaked hay.

High temperature steaming appeared to have the greatest effect on read counts, with the fewest viable bacteria following the HTS treatment. This was supported by the pre-sequencing gel-electrophoresis whereby the steamed samples were more difficult to detect (S2 Fig), and more HTS samples were lost during data analysis due to quality filtering of reads. When considering these findings the loss of samples due to low quality reads should be considered at two levels. The loss of HTS samples did reduce the sample size, however it is likely many of the HTS samples were lost due to the conditions associated with HTS itself. It is plausible that as the hay is exposed to temperatures in excess of 90°C that not only does this process kill a proportion of the viable bacteria but it also causes debris to anneal to bacterial DNA which prevented replication and sequencing. While the process of DNA replication, PCR, uses heat to denature proteins prior to annealing and extension, this is conducted in a controlled manner following DNA extraction, filtering and purification in the presence of the required enzymes and primers. It is therefore plausible that heating bacteria and plant matter together, in this example hay, caused organic debris to act as a PCR inhibitor by annealing to the bacterial DNA thus preventing replication [39].

Previously Moore-Colyer *et al*. [7] identified that HTS significantly reduced the ability to culture bacteria. Many of the microbiota found on the hay are also commonly found in soil. Roux-Michollet *et al*. [40] found that when using steam disinfestation on soil, the soil itself reached 100°C and the authors witnessed a significant reduction in post treatment OTUs in their soil samples. The authors concluded that the temperature of the steam denatured bacterial DNA which led to reduced abundance following steaming. Given the similarities in the bacterial types, use of steam at similar temperatures as Roux-Michollet *et al*. [40] and the likelihood that organic debris acts as a PCR inhibitor, further supports the likelihood that that DNA was denatured. It is also plausible that HTS denatures bacterial DNA on hay and thus maybe the mechanism that leads to a reduction in symptoms of respiratory disease when fed HTS hay [41, 42].

When comparing the viable and non-viable bacteria within the HTS hay samples it appeared that those remaining viable were mainly non-pathogenic soil and normal gut dwelling bacteria. There were *genera* that contain pathogenic bacteria following HTS but the species information was not available and therefore this may have been non-pathogenic. There was also a significant reduction in viable bacteria following HTS, so while there might be some potential pathogens surviving the numbers of these should be small, however this needs to be quantified with qPCR. Of those that were non-viable, several were linked to respiratory disease and dental disease. Specifically *Pseudomonas spp*. and *Stenotrophomonas spp*. that are associated with respiratory disease [43] and *Prevotella nigrescens*, *Prevotella melaninogenica spp*. *and Porphyromonas spp*. which are associated with equine dental disease [44]. These were detected as viable in the dry and soaked samples. Our findings therefore suggest that HTS kills bacteria associated with respiratory and dental disease in horses. Reducing bacteria associated with dental disease has not previously been considered an advantage of HTS hay which has predominantly been focused on improving respiratory health in equids. However reducing any bacterial exposure that pose detrimental to health is advantageous to horses’. This association between reduced viability of bacteria involved in dental disease in horses and hay warrants further exploration in an in *vivo* study environment.

While bacterial viability was reduced by HTS, bacterial viability did not alter bacteria diversity within or between the different pre-feeding regimens, therefore while fewer bacteria survived the HTS treatment the diversity of those remaining did not differ from the dry hay. The effect of soaking did however alter the diversity of bacteria present, reducing the alpha diversity. It is likely that the conditions in the water were favorable for some microbiota which thrived, e.g. proteobacteria, which replaced those that could not survive in those conditions. When considering the bacteria from soaked hay, samples contained viable OTUs associated with both plant and marine pathogens. Previous works have identified that post soak liquor from hay had a BOD ten times greater than raw sewage [10]. Where marine pathogens were detected in the soaked hay but not in the other treatments it is assumed these were transmitted in the water used to soak the hay.

The horse has a particularly sensitive gut microbiome and previous studies have shown that changes in forage, e.g. from grass to grass and haylage, can influence bacterial composition of the gut [13]. Previous studies have also identified that ingestion of Gram-negative bacteria on feedstuffs is accompanied by ingestion of significant levels of lipopolysaccharides (LPS) found on the outer bacterial membrane. LPS can potentially elicit strong and undesirable immune responses in animals [45]. In human studies, increases in abundance of proteobacteria in the microbiome [46] and increases in serum LPS have been observed together in diseased states alongside dysbiosis when compared with healthy individuals [47]. Given that proteobacteria are Gram-negative and therefore carry LPS on the membrane, increasing ingestion of proteobacteria and therefore LPS, should be avoided. Our results showed in increased in proteobacteria from 46% relative abundance on the dry hay to 67% relative abundance after 12 hours soaking. Feeding long soaked hay may therefore risk ingestion of bacteria greater than the safe upper bacterial limit of 20 μg/g for animals [48]. While our findings are not truly quantitative of bacterial numbers they reflect an increase in the abundance of proteobacteria by 21% on forage after long soak periods. Taken with the concept of feedstuffs having the ability to influence the microbiome [12], keeping the diversity of microbiota on forage closer to dry hay e.g. more diverse, rather than reducing and altering bacterial diversity of forage by soaking, may be beneficial for equid gut health. This is an area that warrants further investigation to identify the effect of microbiota on forages on the gastrointestinal microbiota of horses’.

Between the two different types of hay, M and IRG, there were differences in the microbial community profile of the four individual dry hays and how the wetting regimens altered the bacterial community profile for each hay type. However caution needs to be taken when looking at the individual hays due to the limited sample numbers. The IRG grass hays had no unique OTUs whereas the meadow hays both had unique OTUs. Similarly the size of the core community for the IRG hay was smaller than that of the meadow hay for each pre-feeding regimen. This is likely due to the multispecies composition of the meadow hay compared to the monoculture of the ryegrass. This would support the findings of Seguin *et al*. [3] and Moore-Colyer [14] when comparing hygiene of single and multispecies hays.

It is logical that the microbial community profile of hay would be influenced by the location it came from reflecting the soil, local environmental conditions and agricultural practices [4]. However the pre-feeding regimen had a greater effect on bacterial community profile and diversity of microbiota than the type of hay or the location is was sourced from. This also supports the previous culture work whereby HTS had a greater effect on bacterial total viable count than soaking or dry hay sourced from differing locations [7].

There are some limitations to be considered when extrapolating the findings of this study. We used two types of hay, meadow and IRG representing two commonly used hays for horses, however other common hay types such as Timothy and Perennial Rye Grass are not represented here. However, when considering our findings the most significant effect on bacterial community profile was pre-feeding regimen rather than hay type or location. It should also be taken into consideration that some hays had very limited replication due to samples failing to generate data, this could also reflect the effect of the regimens on bacterial DNA. Samples were only from two different geographic locations, it may be beneficial to sample hays from multiple locations. The number of overall samples at the outset may appear limited here, this reflects the cost of bacterial community profiling studies which also reflected the number of locations sampled from and types of hay used. Data from samples treated with PMA are characterised here as viable as it is presumed that non-viable bacteria will not have been amplified after the dye altered the bacteria’s DNA structure. However it is possible that differences in bacterial cell walls may mean that vPCR is not always 100% successful in removing non-viable bacteria and thus false positives can be reported [49, 50]. The use of vPCR can also provide technical challenges in optimizing the technique to gain best results, here we used a previously validated approach that we optimized prior to generating this dataset. One of the proposed limitations of PMA is a limitation to identify live versus dead bacteria where the numbers of dead bacteria are very low [51]. Within our study the differing regimens would have had varying proportions of dead cells present. We speculate the HTS samples would have had the most dead cells following the heat treatment and therefore a reduced risk of a high false positive rate. In comparison the soaked hay samples we know from previous culture work that some bacteria will proliferated in the conditions [7]. Our soaked samples saw the greatest increases in viable OTUs when compared to the total bacteria samples on the same hays, suggesting the PMA treatment was effective. When looking at our findings on the read counts our results suggest that samples that were subject to vPCR across regimens had lower reads than those not subject to vPCR thus suggesting that vPCR was detecting only the viable bacteria within the sample. vPCR is commonly used on specific bacteria in a laboratory setting e.g. *Legionella* or *Salmonella*, in our study there would have been a range of bacteria on each hay and we used vPCR in a field setting. While differing bacteria may have varying susceptibility to PMA the use of vPCR provides a simple way to detect unculturable bacteria on forage samples and our data suggest this was effective in detecting the viable bacterial profile of hay.

When considering the regimens our results show that HTS hays proportionally had the greatest effect on bacterial viability and this could be due to the effect of heat treatment on the bacterial DNA [52], rather than the PMA dye alone on detecting viability if the DNA has annealed to plant debris.

We inferred predicted metabolic function using the PICRUSt package, this tool uses the 16S sequence to predict function. While this methods is commonly undertaken, it uses short 16S sequences which are most reliable in classification to *genera* [53], and only provides a prediction. A more reliable method would be to use full genome sequencing to predict function, this has significant cost implications. However predicting metabolic function gives an insight into the possible roles of the microbiota present. Notwithstanding these limitations we believe that these findings provide a valuable insight into the viability of microbiota on hay fed to horses dry, following soaking or HTS.

## Conclusions

We conclude that high temperature steaming, when compared to soaking or leaving hay dry, significantly reduced bacteria in hays. HTS also kept the diversity of species similar to that of dry hay, whereas soaking reduced diversity though an increase of Gram-negative proteobacteria in the bacterial community profile which may have a negative impact on gastrointestinal health. This study adds to the growing body of evidence that long soaking hay increases the abundance of Gram-negative bacteria which when ingested may have a negative effect on health. Furthermore findings from read counts for microbiota following HTS are suggestive that the mechanism of HTS on microbiota involves denaturing proteins thus reducing the ingestion of viable microbiota. HTS reduced bacteria associated with respiratory and dental disease, while increasing the abundance of bacteria associated with carbohydrate metabolism which is indicative of reduced disease risk in equids.

## Supporting information

S1 Fig Chao1 index of alpha diversity of viable bacteria total bacteria. 1 and 2 denote locations, M and IRG hay type and D, HTS and W pre-feeding regimens. D: M1T n = 3 M1V n = 3, M2T n = 3 M2V n = 3, I1T n = 3 I1V n = 1, I2T n = 3 I2V n = 3; HTS: M1T n = 2 M1V n = 3, M2T n = 3 M2V n = 3, I1T n = 0 I1V n = 0 I2T n = 2 I2V n = 3; W: M1T n = 0 M1V n = 3, M2T n = 3 M2V n = 3, I1T n = 1 I1V n = 2, I2T n = 3, I2V n = 3.
(TIF)

S2 Fig Gel electrophoresis of 18 samples prior to 16S sequencing, DY is dry viable, DN dry total, WY is soaked viable and WN is soaked total and SY is HTS viable SN HTS total bacteria. Positive controls were from a previous study Moore-Colyer *et al*. [14].
(TIF)

S1 Table OTUs present in total bacteria samples not treated with PMA that were not present in viable (PMA) samples, from 95–100% reduction. (PDF)
