# Supplementary figures and images for: The haybiome: Characterising the viable bacterial community profile of four different hays for horses following different pre-feeding regimens
Source: PLoS One. 2020 Nov 17;15(11):e0242373. doi: 10.1371/journal.pone.0242373 (PMC7671497; doi:10.1371/journal.pone.0242373)

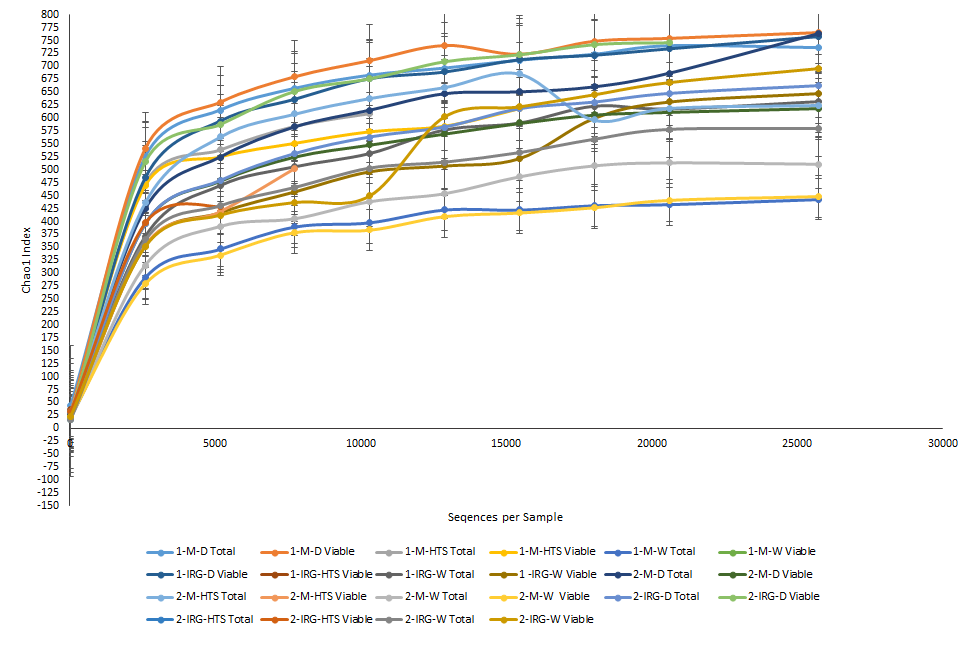

Supplement: S1 Fig — 1 and 2 denote locations, M and IRG hay type and D, HTS and W pre-feeding regimens. D: M1T n = 3 M1V n = 3, M2T n = 3 M2V n = 3, I1T n = 3 I1V n = 1, I2T n = 3 I2V n = 3; HTS: M1T n = 2 M1V n = 3, M2T n = 3 M2V n = 3, I1T n = 0 I1V n = 0 I2T n = 2 I2V n = 3; W: M1T n = 0 M1V n = 3, M2T n = 3 M2V n = 3, I1T n = 1 I1V n = 2, I2T n = 3, I2V n = 3. (TIF) [file pone.0242373.s001.tif]

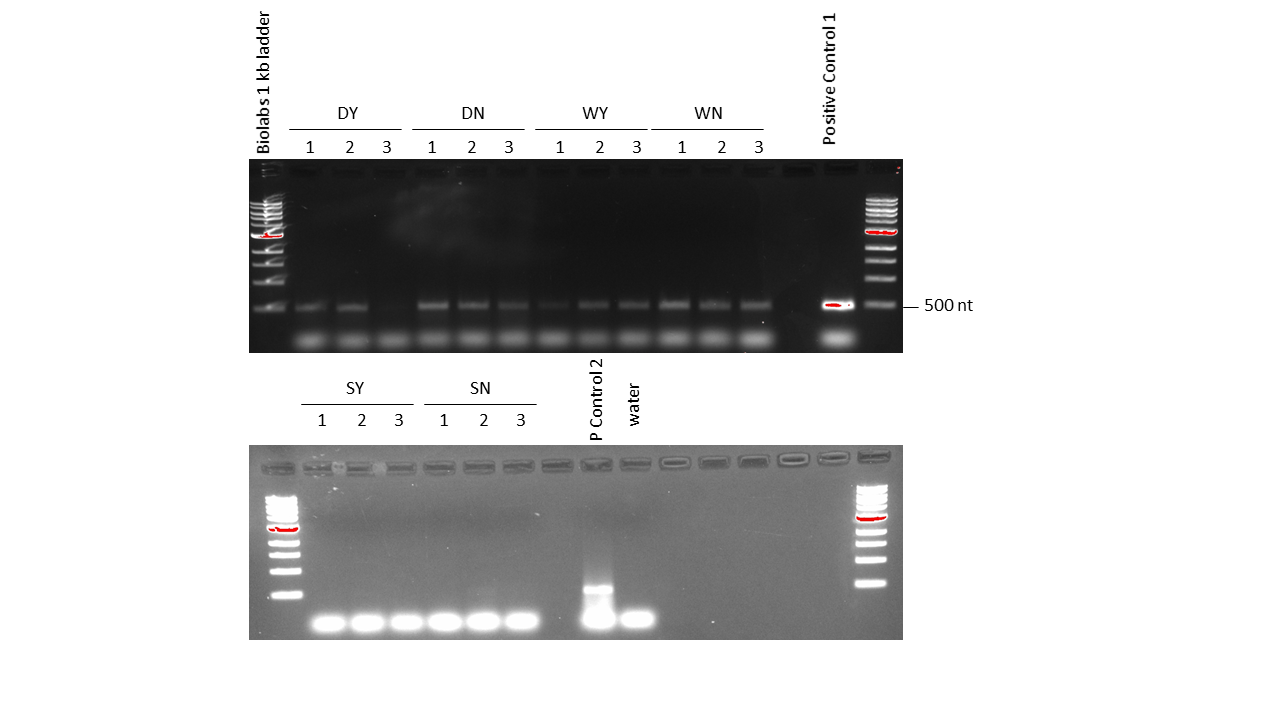

Supplement: S2 Fig — Positive controls were from a previous study Moore-Colyer et al. [14]. (TIF) [file pone.0242373.s002.tif]
