# Supplementary material for: The haybiome: Characterising the viable bacterial community profile of four different hays for horses following different pre-feeding regimens
Source: PLoS One. 2020 Nov 17;15(11):e0242373. doi: 10.1371/journal.pone.0242373 (PMC7671497; doi:10.1371/journal.pone.0242373)
Supplement: S1 Table — (PDF) [file pone.0242373.s003.pdf]

Treatment	O T U	Reduction
Dry	Bacteria|Bacteroidetes|Bacteroidia|Bacteroidales|Porphyromonadaceae|Porphyromonas|	100%
Dry	Bacteria|Bacteroidetes|Bacteroidia|Bacteroidales|Rikenellaceae||	100%
Dry	Bacteria|Bacteroidetes|Bacteroidia|Bacteroidales|Rikenellaceae||	100%
Dry	Bacteria|Bacteroidetes|Flavobacteriia|Flavobacteriales|Flavobacteriaceae|Flavobacterium|	100%
Dry	Bacteria|Firmicutes|Bacilli|Bacillales|Bacillaceae||	100%
Dry	Bacteria|Firmicutes|Bacilli|Bacillales|Bacillaceae|Bacillus|muralis	100%
Dry	Bacteria|Firmicutes|Bacilli|Bacillales|Planococcaceae|Planomicrobium|	100%
Dry	Bacteria|Firmicutes|Bacilli|Bacillales|Staphylococcaceae|Macrococcus|caseolyticus	100%
Dry	Bacteria|Firmicutes|Bacilli|Lactobacillales|Streptococcaceae|Lactococcus|	100%
Dry	Bacteria|Firmicutes|Bacilli|Lactobacillales|Streptococcaceae|Streptococcus|	100%
Dry	Bacteria|Firmicutes|Clostridia|Clostridiales|Ruminococcaceae||	100%
Dry	Bacteria|Proteobacteria|Alphaproteobacteria|Rickettsiales|||	100%
Dry	Bacteria|Proteobacteria|Alphaproteobacteria|Sphingomonadales|Sphingomonadaceae|Novosphingobium|	100%
Dry	Bacteria|Proteobacteria|Betaproteobacteria|Burkholderiales|Comamonadaceae||	100%
Dry	Bacteria|Proteobacteria|Betaproteobacteria|Neisseriales|Neisseriaceae|Neisseria	100%
Dry	Bacteria|Proteobacteria|Betaproteobacteria|Neisseriales|Neisseriaceae|Neisseria|	100%
Dry	Bacteria|Proteobacteria|Betaproteobacteria|Neisseriales|Neisseriaceae|Neisseria|subflava	100%
Dry	Bacteria|Proteobacteria|Gammaproteobacteria|Enterobacteriales|Enterobacteriaceae||	100%
Dry	Bacteria|Proteobacteria|Gammaproteobacteria|Enterobacteriales|Enterobacteriaceae||	100%
Dry	Bacteria|Proteobacteria|Gammaproteobacteria|Enterobacteriales|Enterobacteriaceae||	100%
Dry	Bacteria|Proteobacteria|Gammaproteobacteria|Enterobacteriales|Enterobacteriaceae|Dickeya|	100%
Dry	Bacteria|Proteobacteria|Gammaproteobacteria|Enterobacteriales|Enterobacteriaceae|Erwinia	100%
Dry	Bacteria|Proteobacteria|Gammaproteobacteria|Enterobacteriales|Enterobacteriaceae|Erwinia|	100%
Dry	Bacteria|Proteobacteria|Gammaproteobacteria|Pasteurellales|Pasteurellaceae|Haemophilus|	100%
Dry	Bacteria|Proteobacteria|Gammaproteobacteria|Pasteurellales|Pasteurellaceae|Haemophilus|parainfluenzae	100%
Dry	Bacteria|Proteobacteria|Gammaproteobacteria|Pseudomonadales|Moraxellaceae||	100%
Dry	Bacteria|Proteobacteria|Gammaproteobacteria|Pseudomonadales|Moraxellaceae|Acinetobacter|	100%
Dry	Bacteria|Proteobacteria|Gammaproteobacteria|Pseudomonadales|Moraxellaceae|Acinetobacter|	100%
Dry	Bacteria|Proteobacteria|Gammaproteobacteria|Pseudomonadales|Moraxellaceae|Acinetobacter|	100%

Dry	Bacteria|Proteobacteria|Gammaproteobacteria|Pseudomonadales|Moraxellaceae|Acinetobacter|johnsonii	100%
Dry	Bacteria|Proteobacteria|Gammaproteobacteria|Pseudomonadales|Pseudomonadaceae|Pseudomonas|viridiflava	100%
Dry	Bacteria|Proteobacteria|Gammaproteobacteria|Pseudomonadales|Moraxellaceae|Acinetobacter|	100%
Dry	Bacteria|Proteobacteria|Gammaproteobacteria|Enterobacteriales|Enterobacteriaceae||	100%
Dry	Bacteria|Proteobacteria|Gammaproteobacteria|Pseudomonadales|Moraxellaceae|Acinetobacter|	99%
Dry	Bacteria|Firmicutes|Bacilli|Lactobacillales|Carnobacteriaceae|Carnobacterium|	99%
Dry	Bacteria|Proteobacteria|Gammaproteobacteria|Aeromonadales|Aeromonadaceae||	99%
Dry	Bacteria|Firmicutes|Bacilli|Lactobacillales|Enterococcaceae|Enterococcus|casseliflavus	98%
Dry	Bacteria|Proteobacteria|Gammaproteobacteria|Enterobacteriales|Enterobacteriaceae||	98%
Dry	Bacteria|Proteobacteria|Gammaproteobacteria|Aeromonadales|Aeromonadaceae||	98%
Dry	Bacteria|Proteobacteria|Gammaproteobacteria|Pseudomonadales|Moraxellaceae|Acinetobacter|	98%
Dry	Bacteria|Proteobacteria|Gammaproteobacteria|Enterobacteriales|Enterobacteriaceae	97%
Dry	Bacteria|Proteobacteria|Gammaproteobacteria|Pseudomonadales|Pseudomonadaceae|Pseudomonas|viridiflava	97%
Dry	Bacteria|Proteobacteria|Gammaproteobacteria|Pseudomonadales|Moraxellaceae|Acinetobacter|	97%
Dry	Bacteria|Proteobacteria|Betaproteobacteria|Burkholderiales|Comamonadaceae|Comamonas|	96%
Dry	Bacteria|Proteobacteria|Gammaproteobacteria|Enterobacteriales|Enterobacteriaceae||	96%
Dry	Bacteria|Proteobacteria|Alphaproteobacteria|Sphingomonadales|Sphingomonadaceae|Novosphingobium|	95%
Steam	Bacteria|[Thermi]|Deinococci|Deinococcales|Deinococcaceae|Deinococcus|	100%
Steam	Bacteria|Actinobacteria|Acidimicrobiia|Acidimicrobiales|||	100%
Steam	Bacteria|Actinobacteria|Actinobacteria|Actinomycetales|Actinomycetaceae|Actinomyces|	100%
Steam	Bacteria|Actinobacteria|Actinobacteria|Actinomycetales|Actinosynnemataceae||	100%
Steam	Bacteria|Actinobacteria|Actinobacteria|Actinomycetales|Microbacteriaceae	100%
Steam	Bacteria|Actinobacteria|Actinobacteria|Actinomycetales|Microbacteriaceae||	100%
Steam	Bacteria|Actinobacteria|Actinobacteria|Actinomycetales|Microbacteriaceae|Microbacterium|	100%
Steam	Bacteria|Actinobacteria|Actinobacteria|Actinomycetales|Micromonosporaceae	100%
Steam	Bacteria|Actinobacteria|Actinobacteria|Actinomycetales|Mycobacteriaceae|Mycobacterium|	100%
Steam	Bacteria|Actinobacteria|Actinobacteria|Actinomycetales|Nakamurellaceae||	100%
Steam	Bacteria|Actinobacteria|Actinobacteria|Actinomycetales|Nocardioidaceae||	100%
Steam	Bacteria|Actinobacteria|Actinobacteria|Actinomycetales|Nocardioidaceae|Nocardioides|	100%
Steam	Bacteria|Actinobacteria|Thermoleophilia|Solirubrobacterales|Patulibacteraceae||	100%

Steam	Bacteria|Actinobacteria|Thermoleophilia|Solirubrobacterales|Patulibacteraceae||	100%
Steam	Bacteria|Armatimonadetes|[Fimbriimonadia]|[Fimbriimonadales]|[Fimbriimonadaceae]|Fimbriimonas|	100%
Steam	Bacteria|Bacteroidetes|[Saprospirae]|[Saprospirales]|||	100%
Steam	Bacteria|Bacteroidetes|[Saprospirae]|[Saprospirales]|Chitinophagaceae||	100%
Steam	Bacteria|Bacteroidetes|[Saprospirae]|[Saprospirales]|Chitinophagaceae||	100%
Steam	Bacteria|Bacteroidetes|[Saprospirae]|[Saprospirales]|Chitinophagaceae||	100%
Steam	Bacteria|Bacteroidetes|[Saprospirae]|[Saprospirales]|Chitinophagaceae||	100%
Steam	Bacteria|Bacteroidetes|[Saprospirae]|[Saprospirales]|Chitinophagaceae||	100%
Steam	Bacteria|Bacteroidetes|[Saprospirae]|[Saprospirales]|Chitinophagaceae||	100%
Steam	Bacteria|Bacteroidetes|[Saprospirae]|[Saprospirales]|Chitinophagaceae||	100%
Steam	Bacteria|Bacteroidetes|[Saprospirae]|[Saprospirales]|Chitinophagaceae||	100%
Steam	Bacteria|Bacteroidetes|[Saprospirae]|[Saprospirales]|Chitinophagaceae||	100%
Steam	Bacteria|Bacteroidetes|[Saprospirae]|[Saprospirales]|Chitinophagaceae||	100%
Steam	Bacteria|Bacteroidetes|[Saprospirae]|[Saprospirales]|Chitinophagaceae|Chitinophaga|	100%
Steam	Bacteria|Bacteroidetes|[Saprospirae]|[Saprospirales]|Saprospiraceae||	100%
Steam	Bacteria|Bacteroidetes|Bacteroidia|Bacteroidales|[Paraprevotellaceae]|[Prevotella]|	100%
Steam	Bacteria|Bacteroidetes|Bacteroidia|Bacteroidales|Porphyromonadaceae|Porphyromonas|endodontalis	100%
Steam	Bacteria|Bacteroidetes|Bacteroidia|Bacteroidales|Prevotellaceae|Prevotella|melaninogenica	100%
Steam	Bacteria|Bacteroidetes|Bacteroidia|Bacteroidales|Prevotellaceae|Prevotella|melaninogenica	100%
Steam	Bacteria|Bacteroidetes|Bacteroidia|Bacteroidales|Prevotellaceae|Prevotella|nigrescens	100%
Steam	Bacteria|Bacteroidetes|Bacteroidia|Bacteroidales|Rikenellaceae||	100%
Steam	Bacteria|Bacteroidetes|Cytophagia|Cytophagales|Cytophagaceae||	100%
Steam	Bacteria|Bacteroidetes|Cytophagia|Cytophagales|Cytophagaceae||	100%
Steam	Bacteria|Bacteroidetes|Cytophagia|Cytophagales|Cytophagaceae|Cytophaga|	100%
Steam	Bacteria|Bacteroidetes|Cytophagia|Cytophagales|Cytophagaceae|Cytophaga|	100%
Steam	Bacteria|Bacteroidetes|Cytophagia|Cytophagales|Cytophagaceae|Dyadobacter|	100%
Steam	Bacteria|Bacteroidetes|Cytophagia|Cytophagales|Cytophagaceae|Dyadobacter|	100%
Steam	Bacteria|Bacteroidetes|Cytophagia|Cytophagales|Cytophagaceae|Dyadobacter|	100%
Steam	Bacteria|Bacteroidetes|Cytophagia|Cytophagales|Cytophagaceae|Hymenobacter|	100%
Steam	Bacteria|Bacteroidetes|Cytophagia|Cytophagales|Cytophagaceae|Hymenobacter|	100%

Steam	Bacteria|Bacteroidetes|Cytophagia|Cytophagales|Cytophagaceae|Hymenobacter|	100%
Steam	Bacteria|Bacteroidetes|Cytophagia|Cytophagales|Cytophagaceae|Hymenobacter|	100%
Steam	Bacteria|Bacteroidetes|Cytophagia|Cytophagales|Cytophagaceae|Hymenobacter|	100%
Steam	Bacteria|Bacteroidetes|Cytophagia|Cytophagales|Cytophagaceae|Spirosoma|	100%
Steam	Bacteria|Bacteroidetes|Cytophagia|Cytophagales|Cytophagaceae|Spirosoma|	100%
Steam	Bacteria|Bacteroidetes|Flavobacteriia|Flavobacteriales|[Weeksellaceae]|Chryseobacterium|	100%
Steam	Bacteria|Bacteroidetes|Flavobacteriia|Flavobacteriales|Cryomorphaceae|Fluviicola|	100%
Steam	Bacteria|Bacteroidetes|Flavobacteriia|Flavobacteriales|Cryomorphaceae|Fluviicola|	100%
Steam	Bacteria|Bacteroidetes|Flavobacteriia|Flavobacteriales|Cryomorphaceae|Fluviicola|	100%
Steam	Bacteria|Bacteroidetes|Flavobacteriia|Flavobacteriales|Flavobacteriaceae|Flavobacterium|	100%
Steam	Bacteria|Bacteroidetes|Flavobacteriia|Flavobacteriales|Flavobacteriaceae|Flavobacterium|	100%
Steam	Bacteria|Bacteroidetes|Flavobacteriia|Flavobacteriales|Flavobacteriaceae|Flavobacterium|	100%
Steam	Bacteria|Bacteroidetes|Flavobacteriia|Flavobacteriales|Flavobacteriaceae|Flavobacterium|	100%
Steam	Bacteria|Bacteroidetes|Flavobacteriia|Flavobacteriales|Flavobacteriaceae|Flavobacterium|	100%
Steam	Bacteria|Bacteroidetes|Flavobacteriia|Flavobacteriales|Flavobacteriaceae|Flavobacterium|	100%
Steam	Bacteria|Bacteroidetes|Flavobacteriia|Flavobacteriales|Flavobacteriaceae|Flavobacterium|	100%
Steam	Bacteria|Bacteroidetes|Sphingobacteriia|Sphingobacteriales|||	100%
Steam	Bacteria|Bacteroidetes|Sphingobacteriia|Sphingobacteriales|||	100%
Steam	Bacteria|Bacteroidetes|Sphingobacteriia|Sphingobacteriales|||	100%
Steam	Bacteria|Bacteroidetes|Sphingobacteriia|Sphingobacteriales|Sphingobacteriaceae||	100%
Steam	Bacteria|Bacteroidetes|Sphingobacteriia|Sphingobacteriales|Sphingobacteriaceae||	100%
Steam	Bacteria|Bacteroidetes|Sphingobacteriia|Sphingobacteriales|Sphingobacteriaceae||	100%
Steam	Bacteria|Bacteroidetes|Sphingobacteriia|Sphingobacteriales|Sphingobacteriaceae||	100%
Steam	Bacteria|Bacteroidetes|Sphingobacteriia|Sphingobacteriales|Sphingobacteriaceae||	100%
Steam	Bacteria|Bacteroidetes|Sphingobacteriia|Sphingobacteriales|Sphingobacteriaceae||	100%
Steam	Bacteria|Bacteroidetes|Sphingobacteriia|Sphingobacteriales|Sphingobacteriaceae||	100%
Steam	Bacteria|Bacteroidetes|Sphingobacteriia|Sphingobacteriales|Sphingobacteriaceae||	100%
Steam	Bacteria|Bacteroidetes|Sphingobacteriia|Sphingobacteriales|Sphingobacteriaceae|Pedobacter|	100%
Steam	Bacteria|Bacteroidetes|Sphingobacteriia|Sphingobacteriales|Sphingobacteriaceae|Sphingobacterium|	100%
Steam	Bacteria|Bacteroidetes|Sphingobacteriia|Sphingobacteriales|Sphingobacteriaceae|Sphingobacterium|	100%

Steam	Bacteria|Bacteroidetes|VC2_1_Bac22||||	100%
Steam	Bacteria|Bacteroidetes|VC2_1_Bac22||||	100%
Steam	Bacteria|Bacteroidetes|VC2_1_Bac22||||	100%
Steam	Bacteria|Chloroflexi|Anaerolineae|SBR1031|A4b||	100%
Steam	Bacteria|Cyanobacteria|Chloroplast|Streptophyta|||	100%
Steam	Bacteria|Cyanobacteria|Chloroplast|Streptophyta|||	100%
Steam	Bacteria|Cyanobacteria|ML635J-21||||	100%
Steam	Bacteria|FBP|||||	100%
Steam	Bacteria|FBP|||||	100%
Steam	Bacteria|Fibrobacteres|Fibrobacteria|258ds10|||	100%
Steam	Bacteria|Firmicutes|Clostridia|Clostridiales|||	100%
Steam	Bacteria|Firmicutes|Clostridia|Clostridiales|Veillonellaceae|Veillonella|dispar	100%
Steam	Bacteria|Fusobacteria|Fusobacteriia|Fusobacteriales|Fusobacteriaceae|Fusobacterium|	100%
Steam	Bacteria|Fusobacteria|Fusobacteriia|Fusobacteriales|Leptotrichiaceae|Leptotrichia|	100%
Steam	Bacteria|Proteobacteria|Alphaproteobacteria|BD7-3|||	100%
Steam	Bacteria|Proteobacteria|Alphaproteobacteria|Caulobacterales|Caulobacteraceae||	100%
Steam	Bacteria|Proteobacteria|Alphaproteobacteria|Rhizobiales|Beijerinckiaceae||	100%
Steam	Bacteria|Proteobacteria|Alphaproteobacteria|Rhizobiales|Hyphomicrobiaceae|Devosia|	100%
Steam	Bacteria|Proteobacteria|Alphaproteobacteria|Rhizobiales|Hyphomicrobiaceae|Devosia|	100%
Steam	Bacteria|Proteobacteria|Alphaproteobacteria|Rhizobiales|Hyphomicrobiaceae|Devosia|	100%
Steam	Bacteria|Proteobacteria|Alphaproteobacteria|Rhizobiales|Phyllobacteriaceae	100%
Steam	Bacteria|Proteobacteria|Alphaproteobacteria|Rhizobiales|Rhizobiaceae|Agrobacterium|	100%
Steam	Bacteria|Proteobacteria|Alphaproteobacteria|Rhizobiales|Rhizobiaceae|Rhizobium|	100%
Steam	Bacteria|Proteobacteria|Alphaproteobacteria|Rickettsiales|||	100%
Steam	Bacteria|Proteobacteria|Alphaproteobacteria|Rickettsiales|mitochondria	100%
Steam	Bacteria|Proteobacteria|Alphaproteobacteria|Rickettsiales|mitochondria	100%
Steam	Bacteria|Proteobacteria|Alphaproteobacteria|Rickettsiales|mitochondria	100%
Steam	Bacteria|Proteobacteria|Alphaproteobacteria|Rickettsiales|Rickettsiaceae||	100%
Steam	Bacteria|Proteobacteria|Alphaproteobacteria|Rickettsiales|Rickettsiaceae|Rickettsia|	100%
Steam	Bacteria|Proteobacteria|Alphaproteobacteria|Rickettsiales|Rickettsiaceae|Wolbachia|	100%

Steam	Bacteria|Proteobacteria|Alphaproteobacteria|Sphingomonadales	100%
Steam	Bacteria|Proteobacteria|Alphaproteobacteria|Sphingomonadales|Erythrobacteraceae|Lutibacterium|	100%
Steam	Bacteria|Proteobacteria|Alphaproteobacteria|Sphingomonadales|Sphingomonadaceae	100%
Steam	Bacteria|Proteobacteria|Alphaproteobacteria|Sphingomonadales|Sphingomonadaceae|Novosphingobium|	100%
Steam	Bacteria|Proteobacteria|Alphaproteobacteria|Sphingomonadales|Sphingomonadaceae|Novosphingobium|	100%
Steam	Bacteria|Proteobacteria|Alphaproteobacteria|Sphingomonadales|Sphingomonadaceae|Novosphingobium|	100%
Steam	Bacteria|Proteobacteria|Alphaproteobacteria|Sphingomonadales|Sphingomonadaceae|Sphingobium|	100%
Steam	Bacteria|Proteobacteria|Alphaproteobacteria|Sphingomonadales|Sphingomonadaceae|Sphingobium|	100%
Steam	Bacteria|Proteobacteria|Alphaproteobacteria|Sphingomonadales|Sphingomonadaceae|Sphingomonas|	100%
Steam	Bacteria|Proteobacteria|Alphaproteobacteria|Sphingomonadales|Sphingomonadaceae|Sphingomonas|	100%
Steam	Bacteria|Proteobacteria|Alphaproteobacteria|Sphingomonadales|Sphingomonadaceae|Sphingomonas|	100%
Steam	Bacteria|Proteobacteria|Alphaproteobacteria|Sphingomonadales|Sphingomonadaceae|Sphingomonas|echinoides	100%
Steam	Bacteria|Proteobacteria|Alphaproteobacteria|Sphingomonadales|Sphingomonadaceae|Sphingomonas|echinoides	100%
Steam	Bacteria|Proteobacteria|Alphaproteobacteria|Sphingomonadales|Sphingomonadaceae|Sphingomonas|wittichii	100%
Steam	Bacteria|Proteobacteria|Alphaproteobacteria|Sphingomonadales|Sphingomonadaceae|Sphingomonas|wittichii	100%
Steam	Bacteria|Proteobacteria|Alphaproteobacteria|Sphingomonadales|Sphingomonadaceae|Sphingomonas|wittichii	100%
Steam	Bacteria|Proteobacteria|Alphaproteobacteria|Sphingomonadales|Sphingomonadaceae|Sphingomonas|wittichii	100%
Steam	Bacteria|Proteobacteria|Alphaproteobacteria|Sphingomonadales|Sphingomonadaceae|Sphingomonas|wittichii	100%
Steam	Bacteria|Proteobacteria|Betaproteobacteria|Burkholderiales|Burkholderiaceae||	100%
Steam	Bacteria|Proteobacteria|Betaproteobacteria|Burkholderiales|Comamonadaceae	100%
Steam	Bacteria|Proteobacteria|Betaproteobacteria|Burkholderiales|Comamonadaceae||	100%
Steam	Bacteria|Proteobacteria|Betaproteobacteria|Burkholderiales|Comamonadaceae||	100%
Steam	Bacteria|Proteobacteria|Betaproteobacteria|Burkholderiales|Comamonadaceae||	100%
Steam	Bacteria|Proteobacteria|Betaproteobacteria|Burkholderiales|Comamonadaceae||	100%
Steam	Bacteria|Proteobacteria|Betaproteobacteria|Burkholderiales|Comamonadaceae||	100%
Steam	Bacteria|Proteobacteria|Betaproteobacteria|Burkholderiales|Comamonadaceae||	100%
Steam	Bacteria|Proteobacteria|Betaproteobacteria|Burkholderiales|Comamonadaceae|Methylibium|	100%
Steam	Bacteria|Proteobacteria|Betaproteobacteria|Burkholderiales|Comamonadaceae|Paucibacter|	100%
Steam	Bacteria|Proteobacteria|Betaproteobacteria|Burkholderiales|Oxalobacteraceae||	100%
Steam	Bacteria|Proteobacteria|Betaproteobacteria|Methylophilales|Methylophilaceae||	100%

Steam	Bacteria|Proteobacteria|Betaproteobacteria|Neisseriales|Neisseriaceae|Neisseria	100%
Steam	Bacteria|Proteobacteria|Betaproteobacteria|Procabacteriales|Procabacteriaceae||	100%
Steam	Bacteria|Proteobacteria|Betaproteobacteria|Procabacteriales|Procabacteriaceae||	100%
Steam	Bacteria|Proteobacteria|Deltaproteobacteria|Myxococcales|||	100%
Steam	Bacteria|Proteobacteria|Deltaproteobacteria|Myxococcales|||	100%
Steam	Bacteria|Proteobacteria|Deltaproteobacteria|Myxococcales|||	100%
Steam	Bacteria|Proteobacteria|Deltaproteobacteria|Myxococcales|||	100%
Steam	Bacteria|Proteobacteria|Deltaproteobacteria|Myxococcales|Cystobacterineae||	100%
Steam	Bacteria|Proteobacteria|Gammaproteobacteria|Alteromonadales|Alteromonadaceae|Cellvibrio|	100%
Steam	Bacteria|Proteobacteria|Gammaproteobacteria|Enterobacteriales|Enterobacteriaceae||	100%
Steam	Bacteria|Proteobacteria|Gammaproteobacteria|Pasteurellales|Pasteurellaceae|Haemophilus|	100%
Steam	Bacteria|Proteobacteria|Gammaproteobacteria|Pseudomonadales|Moraxellaceae|Acinetobacter|	100%
Steam	Bacteria|Proteobacteria|Gammaproteobacteria|Pseudomonadales|Pseudomonadaceae|Pseudomonas|	100%
Steam	Bacteria|Proteobacteria|Gammaproteobacteria|Pseudomonadales|Pseudomonadaceae|Pseudomonas|viridiflava	100%
Steam	Bacteria|Proteobacteria|Gammaproteobacteria|Xanthomonadales|Xanthomonadaceae||	100%
Steam	Bacteria|Proteobacteria|Gammaproteobacteria|Xanthomonadales|Xanthomonadaceae||	100%
Steam	Bacteria|Proteobacteria|Gammaproteobacteria|Xanthomonadales|Xanthomonadaceae|Stenotrophomonas	100%
Steam	Bacteria|Proteobacteria|Gammaproteobacteria|Xanthomonadales|Xanthomonadaceae|Stenotrophomonas|maltophilia	100%
Steam	Bacteria|Tenericutes|Mollicutes|Entomoplasmatales|||	100%
Steam	Bacteria|TM7|SC3||||	100%
Steam	Bacteria|Verrucomicrobia|Opitutae|Opitutales|Opitutaceae|Opitutus|	100%
Steam	Bacteria|Verrucomicrobia|Opitutae|Opitutales|Opitutaceae|Opitutus|	100%
Steam	Bacteria|Verrucomicrobia|Verrucomicrobiae|Verrucomicrobiales|Verrucomicrobiaceae|Luteolibacter|	100%
Steam	Unassigned	100%
Steam	Unassigned	100%
Steam	Unassigned	100%
Steam	Bacteria|Cyanobacteria|Chloroplast|Streptophyta|||	100%
Steam	Bacteria|Firmicutes|Bacilli|Lactobacillales|Streptococcaceae|Streptococcus|	99%
Steam	Bacteria|Firmicutes|Bacilli|Lactobacillales|Streptococcaceae|Streptococcus|	99%
Steam	Bacteria|Proteobacteria|Betaproteobacteria|Neisseriales|Neisseriaceae|Neisseria|subflava	99%

Steam	Bacteria|Firmicutes|Bacilli|Lactobacillales|Streptococcaceae|Streptococcus|	98%
Steam	Bacteria|Bacteroidetes|Bacteroidia|Bacteroidales|Porphyromonadaceae|Porphyromonas|	98%
Steam	Bacteria|Proteobacteria|Betaproteobacteria|Neisseriales|Neisseriaceae|Neisseria|	98%
Steam	Bacteria|Firmicutes|Bacilli|Lactobacillales|Streptococcaceae|Streptococcus|	98%
Steam	Bacteria|Proteobacteria|Deltaproteobacteria|Bdellovibrionales|Bacteriovoracaceae||	98%
Steam	Bacteria|Proteobacteria|Gammaproteobacteria|Pasteurellales|Pasteurellaceae|Haemophilus|	98%
Steam	Bacteria|Bacteroidetes|Bacteroidia|Bacteroidales|Prevotellaceae|Prevotella|	98%
Steam	Bacteria|Proteobacteria|Gammaproteobacteria|Enterobacteriales|Enterobacteriaceae||	97%
Steam	Bacteria|Bacteroidetes|Flavobacteriia|Flavobacteriales|Cryomorphaceae|Fluviicola|	97%
Steam	Bacteria|Proteobacteria|Alphaproteobacteria|Sphingomonadales|Sphingomonadaceae|Sphingomonas|	97%
Steam	Bacteria|Bacteroidetes|Flavobacteriia|Flavobacteriales|Flavobacteriaceae|Flavobacterium|succinicans	97%
Steam	Bacteria|Proteobacteria|Alphaproteobacteria|Caulobacterales|Caulobacteraceae||	97%
Steam	Bacteria|Proteobacteria|Gammaproteobacteria|Alteromonadales|[Chromatiaceae]|Rheinheimera|	97%
Steam	Bacteria|Proteobacteria|Gammaproteobacteria|Pasteurellales|Pasteurellaceae|Haemophilus|parainfluenzae	97%
Steam	Bacteria|Firmicutes|Bacilli|Lactobacillales|Streptococcaceae|Streptococcus|	97%
Steam	Bacteria|Proteobacteria|Betaproteobacteria|Burkholderiales|Comamonadaceae|Paucibacter|	96%
Steam	Bacteria|Proteobacteria|Betaproteobacteria|Burkholderiales|Alcaligenaceae|Achromobacter|	96%
Steam	Bacteria|Proteobacteria|Betaproteobacteria|Burkholderiales|Comamonadaceae||	96%
Steam	Bacteria|Bacteroidetes|Flavobacteriia|Flavobacteriales|Cryomorphaceae|Fluviicola|	95%
Steam	Bacteria|Bacteroidetes|Flavobacteriia|Flavobacteriales|Cryomorphaceae|Fluviicola|	95%
Steam	Bacteria|Bacteroidetes|Sphingobacteriia|Sphingobacteriales|||	95%
Steam	Bacteria|Fibrobacteres|Fibrobacteria|258ds10|||	95%
Soak	Bacteria|Actinobacteria|Actinobacteria|Actinomycetales|Microbacteriaceae|Mycetocola|	100%
Soak	Bacteria|Actinobacteria|Actinobacteria|Actinomycetales|Nocardioidaceae||	100%
Soak	Bacteria|Actinobacteria|Actinobacteria|Actinomycetales|Nocardioidaceae||	100%
Soak	Bacteria|Actinobacteria|Actinobacteria|Actinomycetales|Nocardioidaceae|Friedmanniella|	100%
Soak	Bacteria|Actinobacteria|Actinobacteria|Actinomycetales|Tsukamurellaceae|Tsukamurella|	100%
Soak	Bacteria|Actinobacteria|Thermoleophilia|Solirubrobacterales|Patulibacteraceae||	100%
Soak	Bacteria|Bacteroidetes|[Saprospirae]|[Saprospirales]|Chitinophagaceae||	100%
Soak	Bacteria|Bacteroidetes|[Saprospirae]|[Saprospirales]|Chitinophagaceae||	100%

Soak	Bacteria|Bacteroidetes|[Saprospirae]|[Saprospirales]|Chitinophagaceae||	100%
Soak	Bacteria|Bacteroidetes|[Saprospirae]|[Saprospirales]|Chitinophagaceae||	100%
Soak	Bacteria|Bacteroidetes|[Saprospirae]|[Saprospirales]|Chitinophagaceae||	100%
Soak	Bacteria|Bacteroidetes|[Saprospirae]|[Saprospirales]|Chitinophagaceae||	100%
Soak	Bacteria|Bacteroidetes|[Saprospirae]|[Saprospirales]|Chitinophagaceae||	100%
Soak	Bacteria|Bacteroidetes|[Saprospirae]|[Saprospirales]|Saprospiraceae||	100%
Soak	Bacteria|Bacteroidetes|Cytophagia|Cytophagales|Cytophagaceae||	100%
Soak	Bacteria|Bacteroidetes|Cytophagia|Cytophagales|Cytophagaceae|Cytophaga|	100%
Soak	Bacteria|Bacteroidetes|Cytophagia|Cytophagales|Cytophagaceae|Dyadobacter|	100%
Soak	Bacteria|Bacteroidetes|Flavobacteriia|Flavobacteriales|[Weeksellaceae]|Chryseobacterium|	100%
Soak	Bacteria|Bacteroidetes|Flavobacteriia|Flavobacteriales|Cryomorphaceae|Fluviicola|	100%
Soak	Bacteria|Bacteroidetes|Flavobacteriia|Flavobacteriales|Cryomorphaceae|Fluviicola|	100%
Soak	Bacteria|Bacteroidetes|Flavobacteriia|Flavobacteriales|Cryomorphaceae|Fluviicola|	100%
Soak	Bacteria|Bacteroidetes|Flavobacteriia|Flavobacteriales|Flavobacteriaceae|Flavobacterium|	100%
Soak	Bacteria|Bacteroidetes|Flavobacteriia|Flavobacteriales|Flavobacteriaceae|Flavobacterium|	100%
Soak	Bacteria|Bacteroidetes|Sphingobacteriia|Sphingobacteriales|||	100%
Soak	Bacteria|Bacteroidetes|Sphingobacteriia|Sphingobacteriales|||	100%
Soak	Bacteria|Bacteroidetes|Sphingobacteriia|Sphingobacteriales|Sphingobacteriaceae||	100%
Soak	Bacteria|Cyanobacteria|Chloroplast|Streptophyta|||	100%
Soak	Bacteria|Cyanobacteria|ML635J-21||||	100%
Soak	Bacteria|Fibrobacteres|Fibrobacteria|258ds10|||	100%
Soak	Bacteria|Firmicutes|Bacilli|Bacillales|Planococcaceae|Planomicrobium|	100%
Soak	Bacteria|Firmicutes|Bacilli|Lactobacillales	100%
Soak	Bacteria|Firmicutes|Bacilli|Lactobacillales|Carnobacteriaceae|Granulicatella|	100%
Soak	Bacteria|Proteobacteria|Alphaproteobacteria||||	100%
Soak	Bacteria|Proteobacteria|Alphaproteobacteria|Rhizobiales|Hyphomicrobiaceae|Devosia|	100%
Soak	Bacteria|Proteobacteria|Alphaproteobacteria|Sphingomonadales|Sphingomonadaceae|Sphingomonas|wittichii	100%
Soak	Bacteria|Proteobacteria|Betaproteobacteria|Burkholderiales|Oxalobacteraceae||	100%
Soak	Bacteria|Proteobacteria|Betaproteobacteria|Methylophilales|Methylophilaceae||	100%
Soak	Bacteria|Proteobacteria|Gammaproteobacteria|Xanthomonadales|Xanthomonadaceae	100%

Soak	Bacteria|Verrucomicrobia|Opitutae|Opitutales|Opitutaceae|Opitutus|	100%
Soak	Bacteria|Proteobacteria|Alphaproteobacteria|Rickettsiales|Rickettsiaceae|Wolbachia|	98%
Soak	Bacteria|Proteobacteria|Betaproteobacteria|Burkholderiales|Comamonadaceae	98%
Soak	Bacteria|Firmicutes|Bacilli|Bacillales|Bacillaceae|Bacillus|	97%
Soak	Bacteria|Bacteroidetes|Flavobacteriia|Flavobacteriales|[Weeksellaceae]|Chryseobacterium|	96%
Soak	Bacteria|Proteobacteria|Alphaproteobacteria|Rickettsiales|||	96%
Soak	Bacteria|Actinobacteria|Actinobacteria|Actinomycetales|Nocardioidaceae||	95%
Soak	Bacteria|FBP|||||	95%
Soak	Bacteria|Firmicutes|Bacilli|Lactobacillales|Enterococcaceae|Enterococcus|	95%
